# Huge Lymphangioma of the Esophagus Resected by Endoscopic Piecemeal Mucosal Resection

**DOI:** 10.1155/2017/5747560

**Published:** 2017-03-20

**Authors:** Dinghai Luo, Liping Ye, Weidan Wu, Haihong Zheng, Xinli Mao

**Affiliations:** ^1^Department of Gastroenterology, Taizhou Hospital, 150 Ximen Street, Linhai, Zhejiang 317000, China; ^2^Department of Pathology, Taizhou Hospital, 150 Ximen Street, Linhai, Zhejiang 317000, China

## Abstract

We present an unusual case of a 41-year-old male patient with a large lymphangioma of the esophagus. Endoscopy revealed that the structure measured 60 × 10 mm in the mucosa and the submucosa and had a heterogenous echo pattern. The esophageal mass was successfully resected by endoscopic piecemeal mucosal resection. However, most esophageal lymphangiomas that are larger than 2 cm in diameter reported in the literature can be removed only through open surgery. Thus far, we know of no reported cases of endoscopic resection as a treatment for this case.

## 1. Introduction

Lymphangioma of the esophagus is a benign cystic tumor, which is characterized by the presence of chylous or serous fluid within an irregularly dilated lymphatic channel. To date, it has been a rare clinical entity. Most esophageal lymphangiomas reported in the literature are smaller than 2 cm in diameter and can be removed using endoscopy. The present report describes a patient with lymphangioma of the esophagus. Endoscopy after admission revealed a 60 × 10 mm mass in the esophagus, which was then treated by endoscopic piecemeal mucosal resection (EPMR).

## 2. Case Report

A 41-year-old man was admitted to the neighborhood hospital because of dysphagia when eating for the past three months. The gastroscopy showed a submucosal mass in the distal esophagus. He was then transferred to our hospital on August 4, 2011, for further examination and treatment. The personal medical and family histories were unremarkable. There was no abdominal pain, nausea, vomiting, hematemesis, melena, or weight loss, and the physical examination and standard laboratory tests were within normal limits.

**Figure 1:**
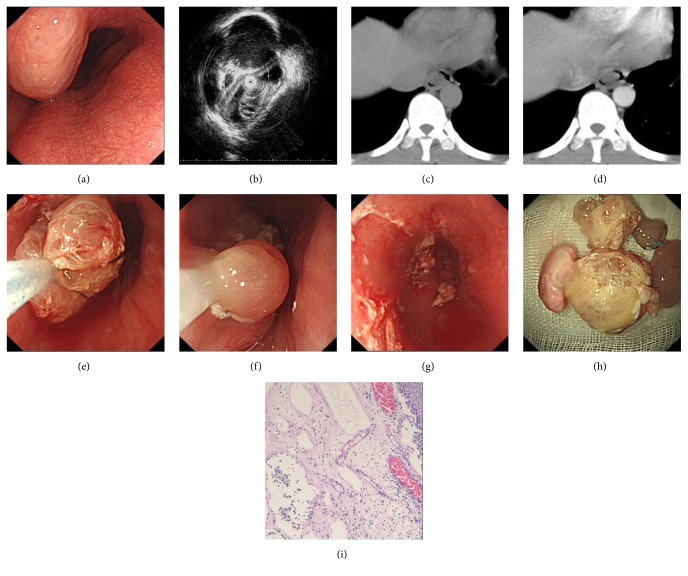
(a) Endoscopic finding. A large, whitish-yellow, translucent mass with a lustrous surface in the mid and distal esophagus, located 32 to 38 cm from the incisors. (b) Endoscopic ultrasound finding. A honeycomb-like hypoechoic structure measuring 60 × 10 mm located in the submucosa with heterogenous echo pattern; the muscularis propria was intact. (c)-(d) Abdominal CT. Contrast-enhanced CT showed the lesion located in the lower esophagus. (e)–(h) The esophageal mass was resected by endoscopic piecemeal mucosal resection (EPMR). (i) Histology of the resected mass showing cystically dilated lymphatics in the surface squamous epithelium and in the submucosa. (hematoxylin and eosin [H&E], magnification ×200).

The endoscopy after admission revealed a large, whitish-yellow, translucent mass with a lustrous surface in the mid and distal esophagus located 32 to 38 cm from the incisors (Figure 1(a)). The mucosa was normal in appearance upon visualization. A later endoscopic biopsy specimen showed mild to moderate dysplasia of the lesion mucosa. Endoscopic ultrasonography (EUS) demonstrated that there was a honeycomb-like hypoechoic structure measuring 60 × 10 mm located in the submucosa with a heterogeneous echo pattern and revealed that the muscularis propria was intact (Figure 1(b)).

Contrast-enhanced CT of the esophagus showed the lesion located in the lower esophagus (Figures 1(c) and 1(d)). The esophageal mass was resected by EPMR on August 5, 2011. We used a needle and inserted it into the tumor body for suction, but there was no outflow of liquid. Then, the esophageal mass was removed using snare electrocautery after injecting the base of the mass with a mixed solution of saline and indigo carmine. The specimen was a cellular-like cystic structure, composed of jelly-like material. The superficial layers of the esophageal wall were clean and contained no significant bleeding (Figures 1(e)–1(h)). The postendoscopic resection course was uneventful, and the patient was well 8 months after the endoscopic resection. Histology of the resected specimen showed cystically dilated lymphatics in the surface squamous epithelium and the submucosa, focally in the muscularis propria with lymphoid fluid in the lumen, and lymphoid aggregates in the stroma between the vessel walls, consistent with lymphangioma (Figure 1(i)). No cysts or stromal tumors were identified. Gastroscopy after eight months showed the original excision wound formed a scar, and rebiopsy confirmed no lesion recurrence. Currently, the patient is still in follow-up.

## 3. Discussion

Lymphangiomas are benign cystic tumors. They are characterized by the presence of chylous or serous fluid within an irregularly dilated lymphatic channel. Lymphangiomas may appear in any organ except the brain, although they commonly develop in the head and neck region, and only 1% are found in the GI tract. Of those that arise in the gut, the majority are found in the colon, followed in order of frequency by the stomach, duodenum, small intestine, and esophagus [1]. The etiology of these tumors is not completely understood [2]. They are thought to be a malformation or sequestration of lymphatic tissue that fails to communicate normally with the lymphatic system [3]. Lymphangioma of the esophagus is a rare clinical entity. It was first reported by Watson-Williams in 1934 [4], and Brady and Milligan were the first persons to diagnose it by endoscopy [5]. Only 16 cases have been reported in the literature to date. The most common presenting symptoms are dysphagia, heartburn, postprandial vomiting, and epigastric pain. It can also present without any symptoms. Endoscopic Ultrasonography is the most important means of diagnosing. Submucosal tumors with multicystic echo pattern within the third layer or submucosa are considered characteristic of lymphangioma [1]. There are generally no difficulties in regard to the pathological diagnosis. Esophageal lymphangioma patients have a good prognosis. Asymptomatic patients do not necessarily need to have the tumor removed, but intervention should be considered when difficulty in swallowing develops or when a mass is suspected to be malignant [6]. Common treatments include surgical resection, endoscopic resection, and carbon dioxide laser treatment. It was reported that treatment with a subcutaneous injection of interferon-2a resulted in a slight improvement in the dysphagia. Although it has side effects [6, 7], the tumor size decreased, and good results were achieved because of its antiproliferative and antiangiogenic effect.

No cases of malignant transformation have been described because lymphangioma is a benign tumor [8], and gastrointestinal lymphangioma of malignant transformation has rarely been reported. Endoscopic resection was applied to the lesions involving the muscularis mucosa and submucosa. However, when the tumor involves the muscularis propria or if a malignant lesion is suspected, thoracic surgery should be performed. For the patient in the present study, preoperative endoscopic ultrasonography demonstrated that the tumor was located in the submucosa, and endoscopic resection achieved good results. Thus, the choice of endoscopic ultrasound has an important role in the diagnosis and treatment of esophageal lymphangioma.

## 4. Conclusions

Our treatment has demonstrated that it is feasible and safe to enucleate lymphangioma even for large lesions. To the best of our knowledge, the endoscopic resection has the advantages of a short operation time, fewer injuries, and a rapid recovery. We believe that EPMR is a safe and sufficient method of therapy for esophageal lymphangioma lesions less than 5 cm in diameter and those located in the mucosa and submucosa.

## Supplementary Material

1. Plain scan CT. Plain scan CT of the esophagus showed the lesion located in the lower esophagus. The CT value is 16 Hu.
2. Enhanced CT. Enhanced CT of the esophagus showed the lesion located in the lower esophagus. The CT value is 38 Hu.
3. EUS. A large, whitish-yellow, translucent mass with a lustrous surface in the mid and distal esophagus, located 32 to 38 cm from the incisors. A honeycomb-like hypoechoic structure measuring 60 × 10 mm located in the submucosa with heterogenous echo pattern; the muscularis propria was intact.
4. EPMR. The esophageal mass was resected by endoscopic piecemeal mucosal resection (EPMR).
5. Pathology. Histology of the resected mass showing cystically dilated lymphatics in the surface squamous epithelium and in the submucosa. (hematoxylin and eosin [H&E], magnification ×200).
