# Supplementary figures and images for: Huge Lymphangioma of the Esophagus Resected by Endoscopic Piecemeal Mucosal Resection
Source: Case Rep Med. 2017 Mar 20;2017:5747560. doi: 10.1155/2017/5747560 (PMC5376932; doi:10.1155/2017/5747560)

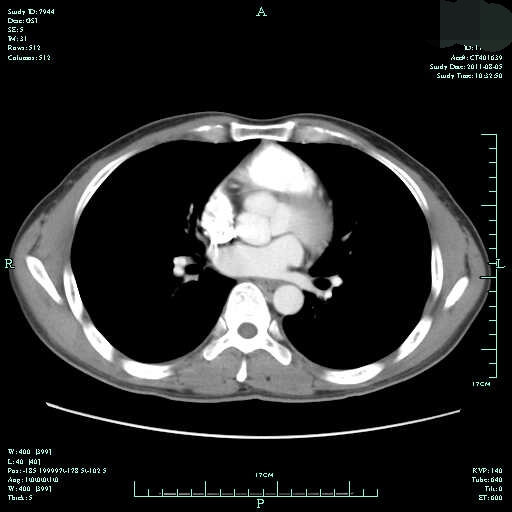

Supplement: Supplementary file 1 — 1. Plain scan CT. Plain scan CT of the esophagus showed the lesion located in the lower esophagus. The CT value is 16 Hu. 2. Enhanced CT. Enhanced CT of the esophagus showed the lesion located in the lower esophagus. The CT value is 38 Hu. 3. EUS. A large, whitish-yellow, translucent mass with a lustrous surface in the mid and distal esophagus, located 32 to 38 cm from the incisors. A honeycomb-like hypoechoic structure measuring 60 × 10 mm located in the submucosa with heterogenous echo pattern; the muscularis propria was intact. 4. EPMR. The esophageal mass was resected by endoscopic piecemeal mucosal resection (EPMR). 5. Pathology. Histology of the resected mass showing cystically dilated lymphatics in the surface squamous epithelium and in the submucosa. (hematoxylin and eosin [H&E], magnification ×200). [file 5747560.f1.zip › EPMR/Enhanced CT/exported0030_看图王.jpg]

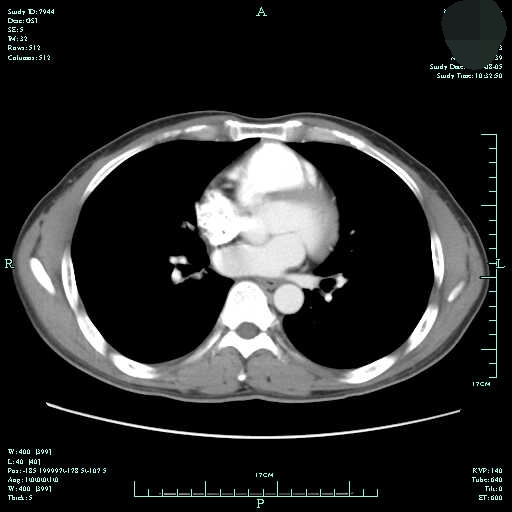

Supplement: Supplementary file 1 — 1. Plain scan CT. Plain scan CT of the esophagus showed the lesion located in the lower esophagus. The CT value is 16 Hu. 2. Enhanced CT. Enhanced CT of the esophagus showed the lesion located in the lower esophagus. The CT value is 38 Hu. 3. EUS. A large, whitish-yellow, translucent mass with a lustrous surface in the mid and distal esophagus, located 32 to 38 cm from the incisors. A honeycomb-like hypoechoic structure measuring 60 × 10 mm located in the submucosa with heterogenous echo pattern; the muscularis propria was intact. 4. EPMR. The esophageal mass was resected by endoscopic piecemeal mucosal resection (EPMR). 5. Pathology. Histology of the resected mass showing cystically dilated lymphatics in the surface squamous epithelium and in the submucosa. (hematoxylin and eosin [H&E], magnification ×200). [file 5747560.f1.zip › EPMR/Enhanced CT/exported0031_看图王.jpg]

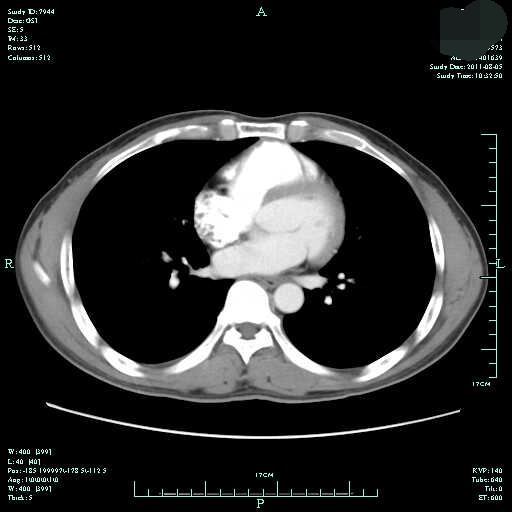

Supplement: Supplementary file 1 — 1. Plain scan CT. Plain scan CT of the esophagus showed the lesion located in the lower esophagus. The CT value is 16 Hu. 2. Enhanced CT. Enhanced CT of the esophagus showed the lesion located in the lower esophagus. The CT value is 38 Hu. 3. EUS. A large, whitish-yellow, translucent mass with a lustrous surface in the mid and distal esophagus, located 32 to 38 cm from the incisors. A honeycomb-like hypoechoic structure measuring 60 × 10 mm located in the submucosa with heterogenous echo pattern; the muscularis propria was intact. 4. EPMR. The esophageal mass was resected by endoscopic piecemeal mucosal resection (EPMR). 5. Pathology. Histology of the resected mass showing cystically dilated lymphatics in the surface squamous epithelium and in the submucosa. (hematoxylin and eosin [H&E], magnification ×200). [file 5747560.f1.zip › EPMR/Enhanced CT/exported0032_看图王.jpg]

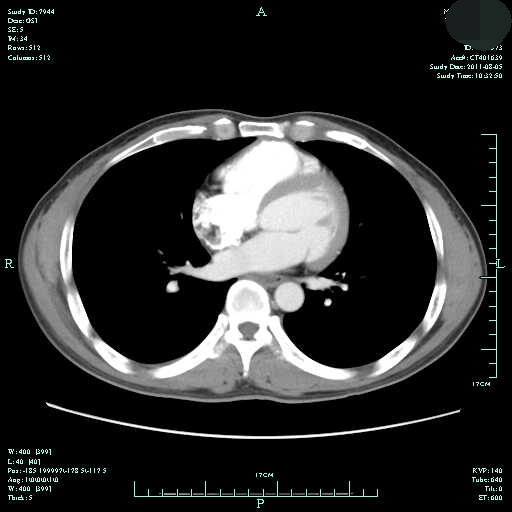

Supplement: Supplementary file 1 — 1. Plain scan CT. Plain scan CT of the esophagus showed the lesion located in the lower esophagus. The CT value is 16 Hu. 2. Enhanced CT. Enhanced CT of the esophagus showed the lesion located in the lower esophagus. The CT value is 38 Hu. 3. EUS. A large, whitish-yellow, translucent mass with a lustrous surface in the mid and distal esophagus, located 32 to 38 cm from the incisors. A honeycomb-like hypoechoic structure measuring 60 × 10 mm located in the submucosa with heterogenous echo pattern; the muscularis propria was intact. 4. EPMR. The esophageal mass was resected by endoscopic piecemeal mucosal resection (EPMR). 5. Pathology. Histology of the resected mass showing cystically dilated lymphatics in the surface squamous epithelium and in the submucosa. (hematoxylin and eosin [H&E], magnification ×200). [file 5747560.f1.zip › EPMR/Enhanced CT/exported0033_看图王.jpg]

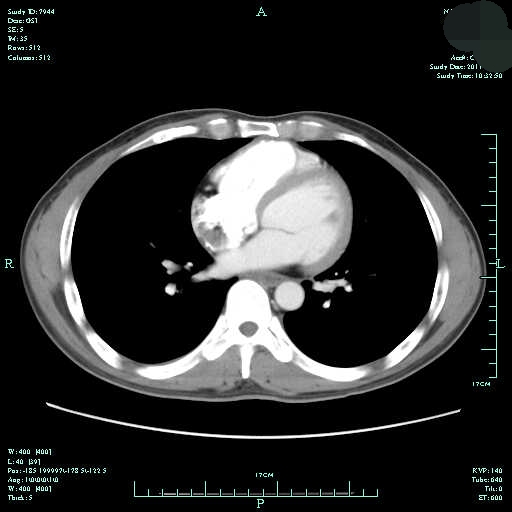

Supplement: Supplementary file 1 — 1. Plain scan CT. Plain scan CT of the esophagus showed the lesion located in the lower esophagus. The CT value is 16 Hu. 2. Enhanced CT. Enhanced CT of the esophagus showed the lesion located in the lower esophagus. The CT value is 38 Hu. 3. EUS. A large, whitish-yellow, translucent mass with a lustrous surface in the mid and distal esophagus, located 32 to 38 cm from the incisors. A honeycomb-like hypoechoic structure measuring 60 × 10 mm located in the submucosa with heterogenous echo pattern; the muscularis propria was intact. 4. EPMR. The esophageal mass was resected by endoscopic piecemeal mucosal resection (EPMR). 5. Pathology. Histology of the resected mass showing cystically dilated lymphatics in the surface squamous epithelium and in the submucosa. (hematoxylin and eosin [H&E], magnification ×200). [file 5747560.f1.zip › EPMR/Enhanced CT/exported0034_看图王.jpg]

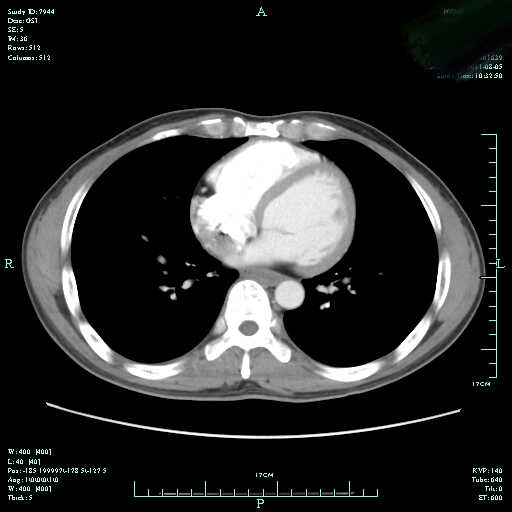

Supplement: Supplementary file 1 — 1. Plain scan CT. Plain scan CT of the esophagus showed the lesion located in the lower esophagus. The CT value is 16 Hu. 2. Enhanced CT. Enhanced CT of the esophagus showed the lesion located in the lower esophagus. The CT value is 38 Hu. 3. EUS. A large, whitish-yellow, translucent mass with a lustrous surface in the mid and distal esophagus, located 32 to 38 cm from the incisors. A honeycomb-like hypoechoic structure measuring 60 × 10 mm located in the submucosa with heterogenous echo pattern; the muscularis propria was intact. 4. EPMR. The esophageal mass was resected by endoscopic piecemeal mucosal resection (EPMR). 5. Pathology. Histology of the resected mass showing cystically dilated lymphatics in the surface squamous epithelium and in the submucosa. (hematoxylin and eosin [H&E], magnification ×200). [file 5747560.f1.zip › EPMR/Enhanced CT/exported0035_看图王.jpg]

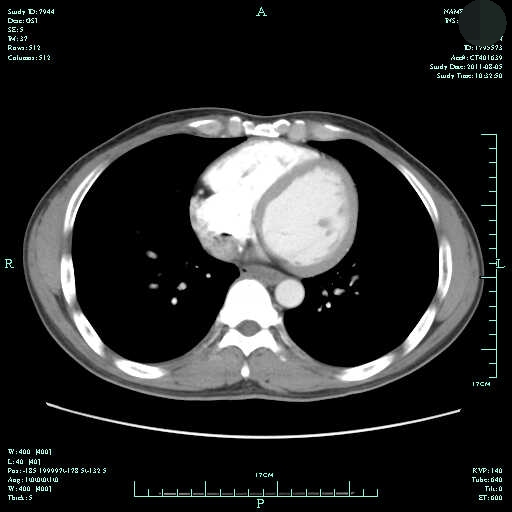

Supplement: Supplementary file 1 — 1. Plain scan CT. Plain scan CT of the esophagus showed the lesion located in the lower esophagus. The CT value is 16 Hu. 2. Enhanced CT. Enhanced CT of the esophagus showed the lesion located in the lower esophagus. The CT value is 38 Hu. 3. EUS. A large, whitish-yellow, translucent mass with a lustrous surface in the mid and distal esophagus, located 32 to 38 cm from the incisors. A honeycomb-like hypoechoic structure measuring 60 × 10 mm located in the submucosa with heterogenous echo pattern; the muscularis propria was intact. 4. EPMR. The esophageal mass was resected by endoscopic piecemeal mucosal resection (EPMR). 5. Pathology. Histology of the resected mass showing cystically dilated lymphatics in the surface squamous epithelium and in the submucosa. (hematoxylin and eosin [H&E], magnification ×200). [file 5747560.f1.zip › EPMR/Enhanced CT/exported0036_看图王.jpg]

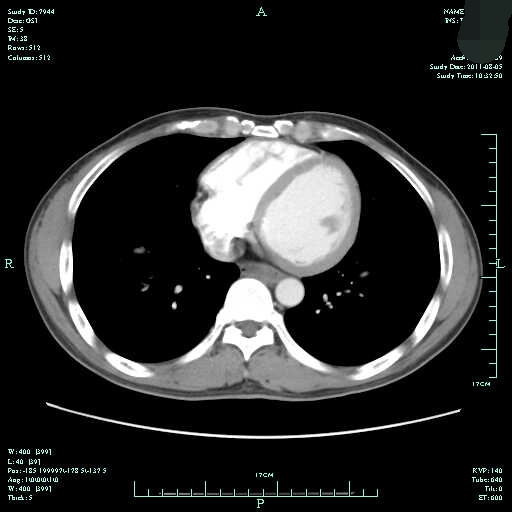

Supplement: Supplementary file 1 — 1. Plain scan CT. Plain scan CT of the esophagus showed the lesion located in the lower esophagus. The CT value is 16 Hu. 2. Enhanced CT. Enhanced CT of the esophagus showed the lesion located in the lower esophagus. The CT value is 38 Hu. 3. EUS. A large, whitish-yellow, translucent mass with a lustrous surface in the mid and distal esophagus, located 32 to 38 cm from the incisors. A honeycomb-like hypoechoic structure measuring 60 × 10 mm located in the submucosa with heterogenous echo pattern; the muscularis propria was intact. 4. EPMR. The esophageal mass was resected by endoscopic piecemeal mucosal resection (EPMR). 5. Pathology. Histology of the resected mass showing cystically dilated lymphatics in the surface squamous epithelium and in the submucosa. (hematoxylin and eosin [H&E], magnification ×200). [file 5747560.f1.zip › EPMR/Enhanced CT/exported0037_看图王.jpg]

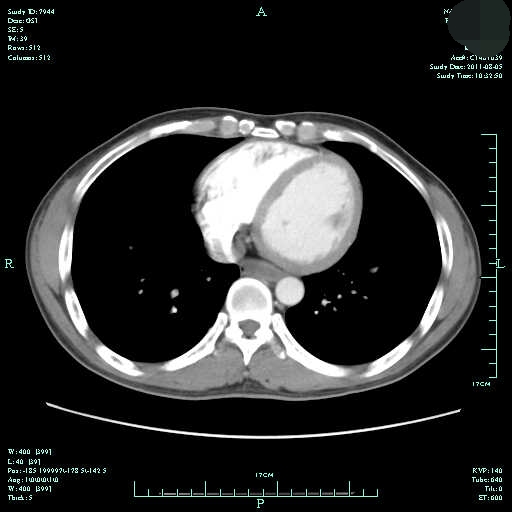

Supplement: Supplementary file 1 — 1. Plain scan CT. Plain scan CT of the esophagus showed the lesion located in the lower esophagus. The CT value is 16 Hu. 2. Enhanced CT. Enhanced CT of the esophagus showed the lesion located in the lower esophagus. The CT value is 38 Hu. 3. EUS. A large, whitish-yellow, translucent mass with a lustrous surface in the mid and distal esophagus, located 32 to 38 cm from the incisors. A honeycomb-like hypoechoic structure measuring 60 × 10 mm located in the submucosa with heterogenous echo pattern; the muscularis propria was intact. 4. EPMR. The esophageal mass was resected by endoscopic piecemeal mucosal resection (EPMR). 5. Pathology. Histology of the resected mass showing cystically dilated lymphatics in the surface squamous epithelium and in the submucosa. (hematoxylin and eosin [H&E], magnification ×200). [file 5747560.f1.zip › EPMR/Enhanced CT/exported0038_看图王.jpg]

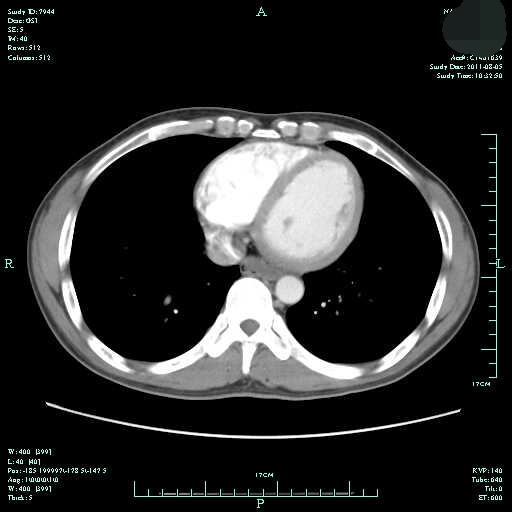

Supplement: Supplementary file 1 — 1. Plain scan CT. Plain scan CT of the esophagus showed the lesion located in the lower esophagus. The CT value is 16 Hu. 2. Enhanced CT. Enhanced CT of the esophagus showed the lesion located in the lower esophagus. The CT value is 38 Hu. 3. EUS. A large, whitish-yellow, translucent mass with a lustrous surface in the mid and distal esophagus, located 32 to 38 cm from the incisors. A honeycomb-like hypoechoic structure measuring 60 × 10 mm located in the submucosa with heterogenous echo pattern; the muscularis propria was intact. 4. EPMR. The esophageal mass was resected by endoscopic piecemeal mucosal resection (EPMR). 5. Pathology. Histology of the resected mass showing cystically dilated lymphatics in the surface squamous epithelium and in the submucosa. (hematoxylin and eosin [H&E], magnification ×200). [file 5747560.f1.zip › EPMR/Enhanced CT/exported0039_看图王.jpg]

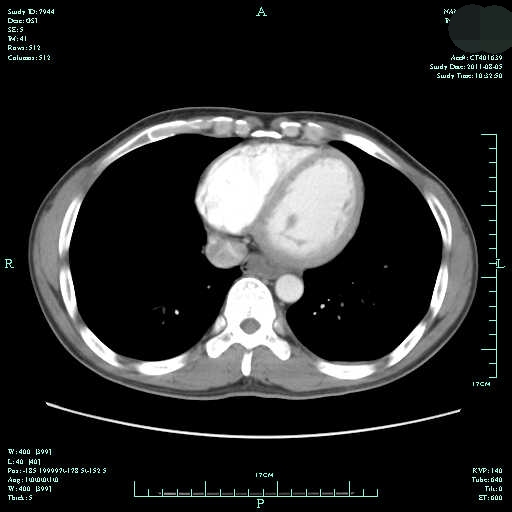

Supplement: Supplementary file 1 — 1. Plain scan CT. Plain scan CT of the esophagus showed the lesion located in the lower esophagus. The CT value is 16 Hu. 2. Enhanced CT. Enhanced CT of the esophagus showed the lesion located in the lower esophagus. The CT value is 38 Hu. 3. EUS. A large, whitish-yellow, translucent mass with a lustrous surface in the mid and distal esophagus, located 32 to 38 cm from the incisors. A honeycomb-like hypoechoic structure measuring 60 × 10 mm located in the submucosa with heterogenous echo pattern; the muscularis propria was intact. 4. EPMR. The esophageal mass was resected by endoscopic piecemeal mucosal resection (EPMR). 5. Pathology. Histology of the resected mass showing cystically dilated lymphatics in the surface squamous epithelium and in the submucosa. (hematoxylin and eosin [H&E], magnification ×200). [file 5747560.f1.zip › EPMR/Enhanced CT/exported0040_看图王.jpg]

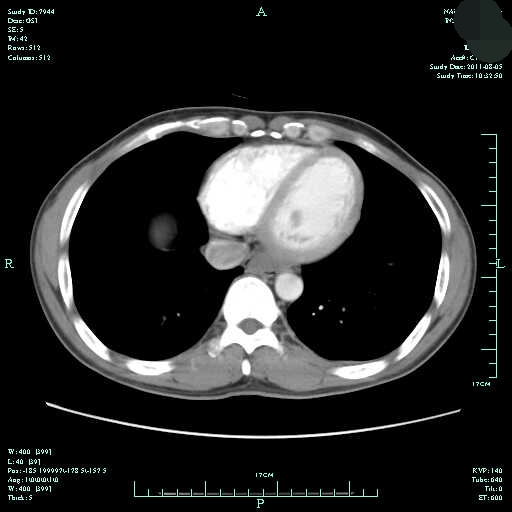

Supplement: Supplementary file 1 — 1. Plain scan CT. Plain scan CT of the esophagus showed the lesion located in the lower esophagus. The CT value is 16 Hu. 2. Enhanced CT. Enhanced CT of the esophagus showed the lesion located in the lower esophagus. The CT value is 38 Hu. 3. EUS. A large, whitish-yellow, translucent mass with a lustrous surface in the mid and distal esophagus, located 32 to 38 cm from the incisors. A honeycomb-like hypoechoic structure measuring 60 × 10 mm located in the submucosa with heterogenous echo pattern; the muscularis propria was intact. 4. EPMR. The esophageal mass was resected by endoscopic piecemeal mucosal resection (EPMR). 5. Pathology. Histology of the resected mass showing cystically dilated lymphatics in the surface squamous epithelium and in the submucosa. (hematoxylin and eosin [H&E], magnification ×200). [file 5747560.f1.zip › EPMR/Enhanced CT/exported0041_看图王.jpg]

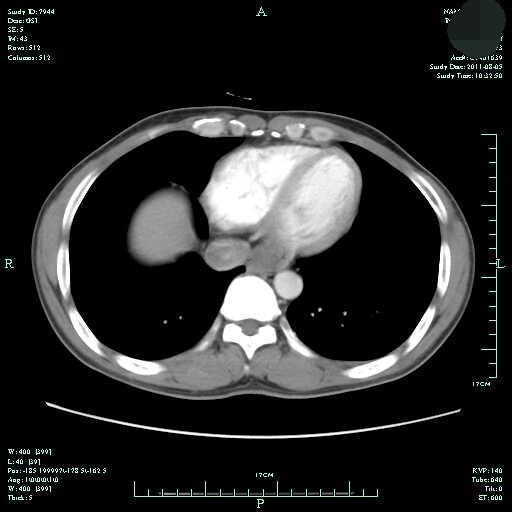

Supplement: Supplementary file 1 — 1. Plain scan CT. Plain scan CT of the esophagus showed the lesion located in the lower esophagus. The CT value is 16 Hu. 2. Enhanced CT. Enhanced CT of the esophagus showed the lesion located in the lower esophagus. The CT value is 38 Hu. 3. EUS. A large, whitish-yellow, translucent mass with a lustrous surface in the mid and distal esophagus, located 32 to 38 cm from the incisors. A honeycomb-like hypoechoic structure measuring 60 × 10 mm located in the submucosa with heterogenous echo pattern; the muscularis propria was intact. 4. EPMR. The esophageal mass was resected by endoscopic piecemeal mucosal resection (EPMR). 5. Pathology. Histology of the resected mass showing cystically dilated lymphatics in the surface squamous epithelium and in the submucosa. (hematoxylin and eosin [H&E], magnification ×200). [file 5747560.f1.zip › EPMR/Enhanced CT/exported0042_看图王.jpg]

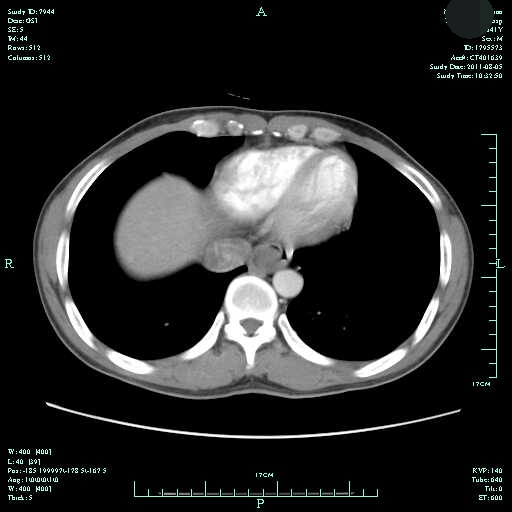

Supplement: Supplementary file 1 — 1. Plain scan CT. Plain scan CT of the esophagus showed the lesion located in the lower esophagus. The CT value is 16 Hu. 2. Enhanced CT. Enhanced CT of the esophagus showed the lesion located in the lower esophagus. The CT value is 38 Hu. 3. EUS. A large, whitish-yellow, translucent mass with a lustrous surface in the mid and distal esophagus, located 32 to 38 cm from the incisors. A honeycomb-like hypoechoic structure measuring 60 × 10 mm located in the submucosa with heterogenous echo pattern; the muscularis propria was intact. 4. EPMR. The esophageal mass was resected by endoscopic piecemeal mucosal resection (EPMR). 5. Pathology. Histology of the resected mass showing cystically dilated lymphatics in the surface squamous epithelium and in the submucosa. (hematoxylin and eosin [H&E], magnification ×200). [file 5747560.f1.zip › EPMR/Enhanced CT/exported0043_看图王.jpg]

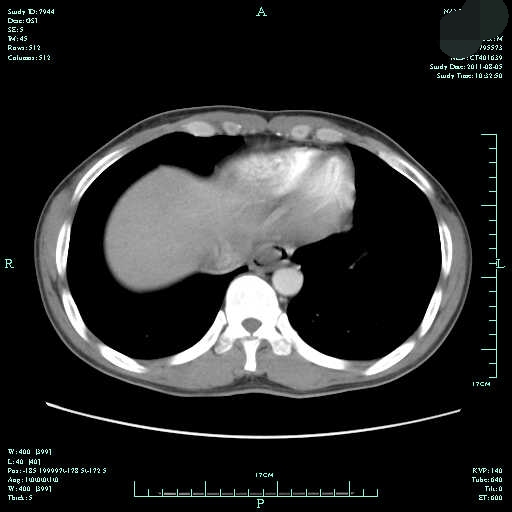

Supplement: Supplementary file 1 — 1. Plain scan CT. Plain scan CT of the esophagus showed the lesion located in the lower esophagus. The CT value is 16 Hu. 2. Enhanced CT. Enhanced CT of the esophagus showed the lesion located in the lower esophagus. The CT value is 38 Hu. 3. EUS. A large, whitish-yellow, translucent mass with a lustrous surface in the mid and distal esophagus, located 32 to 38 cm from the incisors. A honeycomb-like hypoechoic structure measuring 60 × 10 mm located in the submucosa with heterogenous echo pattern; the muscularis propria was intact. 4. EPMR. The esophageal mass was resected by endoscopic piecemeal mucosal resection (EPMR). 5. Pathology. Histology of the resected mass showing cystically dilated lymphatics in the surface squamous epithelium and in the submucosa. (hematoxylin and eosin [H&E], magnification ×200). [file 5747560.f1.zip › EPMR/Enhanced CT/exported0044_看图王.jpg]

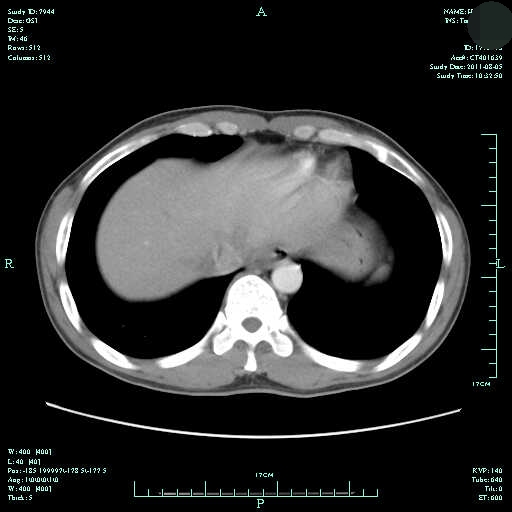

Supplement: Supplementary file 1 — 1. Plain scan CT. Plain scan CT of the esophagus showed the lesion located in the lower esophagus. The CT value is 16 Hu. 2. Enhanced CT. Enhanced CT of the esophagus showed the lesion located in the lower esophagus. The CT value is 38 Hu. 3. EUS. A large, whitish-yellow, translucent mass with a lustrous surface in the mid and distal esophagus, located 32 to 38 cm from the incisors. A honeycomb-like hypoechoic structure measuring 60 × 10 mm located in the submucosa with heterogenous echo pattern; the muscularis propria was intact. 4. EPMR. The esophageal mass was resected by endoscopic piecemeal mucosal resection (EPMR). 5. Pathology. Histology of the resected mass showing cystically dilated lymphatics in the surface squamous epithelium and in the submucosa. (hematoxylin and eosin [H&E], magnification ×200). [file 5747560.f1.zip › EPMR/Enhanced CT/exported0045_看图王.jpg]

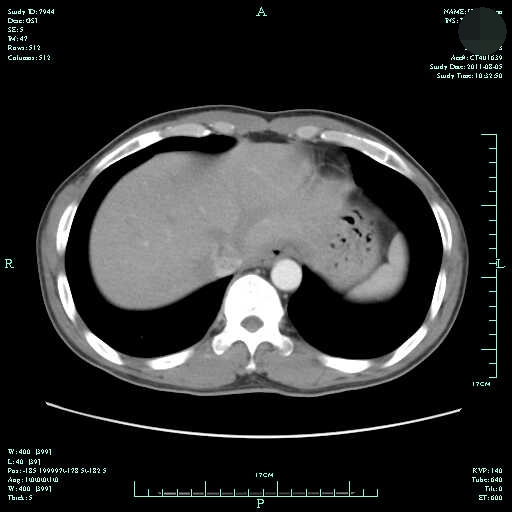

Supplement: Supplementary file 1 — 1. Plain scan CT. Plain scan CT of the esophagus showed the lesion located in the lower esophagus. The CT value is 16 Hu. 2. Enhanced CT. Enhanced CT of the esophagus showed the lesion located in the lower esophagus. The CT value is 38 Hu. 3. EUS. A large, whitish-yellow, translucent mass with a lustrous surface in the mid and distal esophagus, located 32 to 38 cm from the incisors. A honeycomb-like hypoechoic structure measuring 60 × 10 mm located in the submucosa with heterogenous echo pattern; the muscularis propria was intact. 4. EPMR. The esophageal mass was resected by endoscopic piecemeal mucosal resection (EPMR). 5. Pathology. Histology of the resected mass showing cystically dilated lymphatics in the surface squamous epithelium and in the submucosa. (hematoxylin and eosin [H&E], magnification ×200). [file 5747560.f1.zip › EPMR/Enhanced CT/exported0046_看图王.jpg]

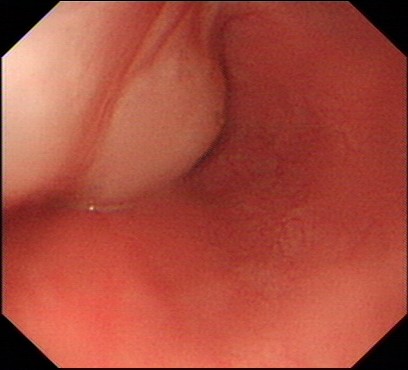

Supplement: Supplementary file 1 — 1. Plain scan CT. Plain scan CT of the esophagus showed the lesion located in the lower esophagus. The CT value is 16 Hu. 2. Enhanced CT. Enhanced CT of the esophagus showed the lesion located in the lower esophagus. The CT value is 38 Hu. 3. EUS. A large, whitish-yellow, translucent mass with a lustrous surface in the mid and distal esophagus, located 32 to 38 cm from the incisors. A honeycomb-like hypoechoic structure measuring 60 × 10 mm located in the submucosa with heterogenous echo pattern; the muscularis propria was intact. 4. EPMR. The esophageal mass was resected by endoscopic piecemeal mucosal resection (EPMR). 5. Pathology. Histology of the resected mass showing cystically dilated lymphatics in the surface squamous epithelium and in the submucosa. (hematoxylin and eosin [H&E], magnification ×200). [file 5747560.f1.zip › EPMR/EPMR/_0001.JPG]

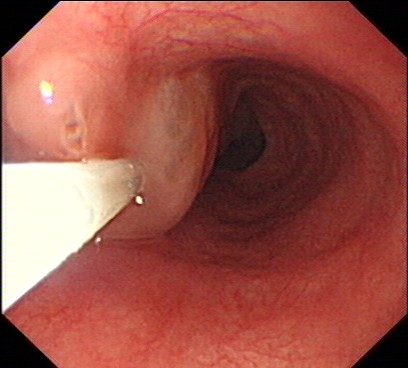

Supplement: Supplementary file 1 — 1. Plain scan CT. Plain scan CT of the esophagus showed the lesion located in the lower esophagus. The CT value is 16 Hu. 2. Enhanced CT. Enhanced CT of the esophagus showed the lesion located in the lower esophagus. The CT value is 38 Hu. 3. EUS. A large, whitish-yellow, translucent mass with a lustrous surface in the mid and distal esophagus, located 32 to 38 cm from the incisors. A honeycomb-like hypoechoic structure measuring 60 × 10 mm located in the submucosa with heterogenous echo pattern; the muscularis propria was intact. 4. EPMR. The esophageal mass was resected by endoscopic piecemeal mucosal resection (EPMR). 5. Pathology. Histology of the resected mass showing cystically dilated lymphatics in the surface squamous epithelium and in the submucosa. (hematoxylin and eosin [H&E], magnification ×200). [file 5747560.f1.zip › EPMR/EPMR/_0002.JPG]

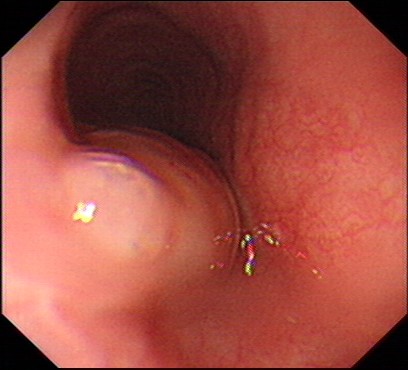

Supplement: Supplementary file 1 — 1. Plain scan CT. Plain scan CT of the esophagus showed the lesion located in the lower esophagus. The CT value is 16 Hu. 2. Enhanced CT. Enhanced CT of the esophagus showed the lesion located in the lower esophagus. The CT value is 38 Hu. 3. EUS. A large, whitish-yellow, translucent mass with a lustrous surface in the mid and distal esophagus, located 32 to 38 cm from the incisors. A honeycomb-like hypoechoic structure measuring 60 × 10 mm located in the submucosa with heterogenous echo pattern; the muscularis propria was intact. 4. EPMR. The esophageal mass was resected by endoscopic piecemeal mucosal resection (EPMR). 5. Pathology. Histology of the resected mass showing cystically dilated lymphatics in the surface squamous epithelium and in the submucosa. (hematoxylin and eosin [H&E], magnification ×200). [file 5747560.f1.zip › EPMR/EPMR/_0003.JPG]

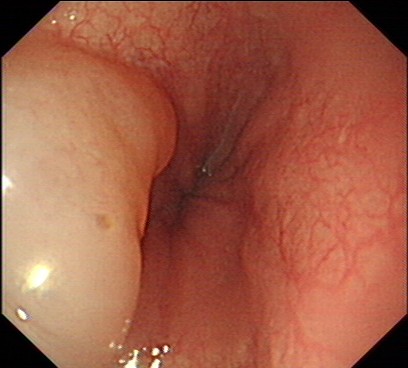

Supplement: Supplementary file 1 — 1. Plain scan CT. Plain scan CT of the esophagus showed the lesion located in the lower esophagus. The CT value is 16 Hu. 2. Enhanced CT. Enhanced CT of the esophagus showed the lesion located in the lower esophagus. The CT value is 38 Hu. 3. EUS. A large, whitish-yellow, translucent mass with a lustrous surface in the mid and distal esophagus, located 32 to 38 cm from the incisors. A honeycomb-like hypoechoic structure measuring 60 × 10 mm located in the submucosa with heterogenous echo pattern; the muscularis propria was intact. 4. EPMR. The esophageal mass was resected by endoscopic piecemeal mucosal resection (EPMR). 5. Pathology. Histology of the resected mass showing cystically dilated lymphatics in the surface squamous epithelium and in the submucosa. (hematoxylin and eosin [H&E], magnification ×200). [file 5747560.f1.zip › EPMR/EPMR/_0004.JPG]

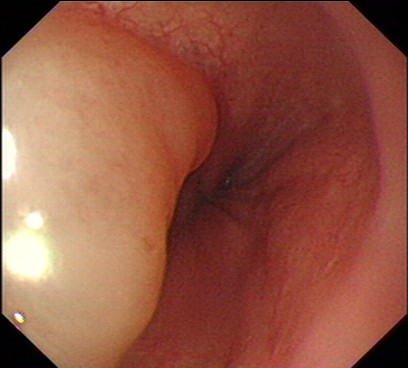

Supplement: Supplementary file 1 — 1. Plain scan CT. Plain scan CT of the esophagus showed the lesion located in the lower esophagus. The CT value is 16 Hu. 2. Enhanced CT. Enhanced CT of the esophagus showed the lesion located in the lower esophagus. The CT value is 38 Hu. 3. EUS. A large, whitish-yellow, translucent mass with a lustrous surface in the mid and distal esophagus, located 32 to 38 cm from the incisors. A honeycomb-like hypoechoic structure measuring 60 × 10 mm located in the submucosa with heterogenous echo pattern; the muscularis propria was intact. 4. EPMR. The esophageal mass was resected by endoscopic piecemeal mucosal resection (EPMR). 5. Pathology. Histology of the resected mass showing cystically dilated lymphatics in the surface squamous epithelium and in the submucosa. (hematoxylin and eosin [H&E], magnification ×200). [file 5747560.f1.zip › EPMR/EPMR/_0005.JPG]

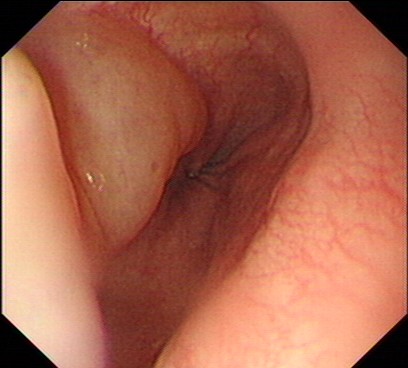

Supplement: Supplementary file 1 — 1. Plain scan CT. Plain scan CT of the esophagus showed the lesion located in the lower esophagus. The CT value is 16 Hu. 2. Enhanced CT. Enhanced CT of the esophagus showed the lesion located in the lower esophagus. The CT value is 38 Hu. 3. EUS. A large, whitish-yellow, translucent mass with a lustrous surface in the mid and distal esophagus, located 32 to 38 cm from the incisors. A honeycomb-like hypoechoic structure measuring 60 × 10 mm located in the submucosa with heterogenous echo pattern; the muscularis propria was intact. 4. EPMR. The esophageal mass was resected by endoscopic piecemeal mucosal resection (EPMR). 5. Pathology. Histology of the resected mass showing cystically dilated lymphatics in the surface squamous epithelium and in the submucosa. (hematoxylin and eosin [H&E], magnification ×200). [file 5747560.f1.zip › EPMR/EPMR/_0006.JPG]

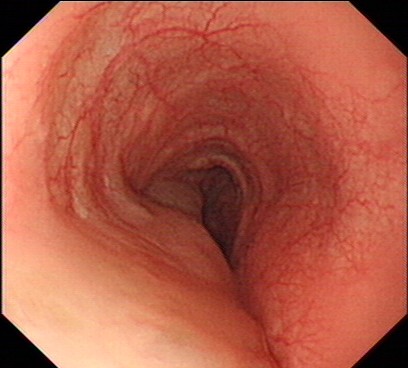

Supplement: Supplementary file 1 — 1. Plain scan CT. Plain scan CT of the esophagus showed the lesion located in the lower esophagus. The CT value is 16 Hu. 2. Enhanced CT. Enhanced CT of the esophagus showed the lesion located in the lower esophagus. The CT value is 38 Hu. 3. EUS. A large, whitish-yellow, translucent mass with a lustrous surface in the mid and distal esophagus, located 32 to 38 cm from the incisors. A honeycomb-like hypoechoic structure measuring 60 × 10 mm located in the submucosa with heterogenous echo pattern; the muscularis propria was intact. 4. EPMR. The esophageal mass was resected by endoscopic piecemeal mucosal resection (EPMR). 5. Pathology. Histology of the resected mass showing cystically dilated lymphatics in the surface squamous epithelium and in the submucosa. (hematoxylin and eosin [H&E], magnification ×200). [file 5747560.f1.zip › EPMR/EPMR/_0007.JPG]

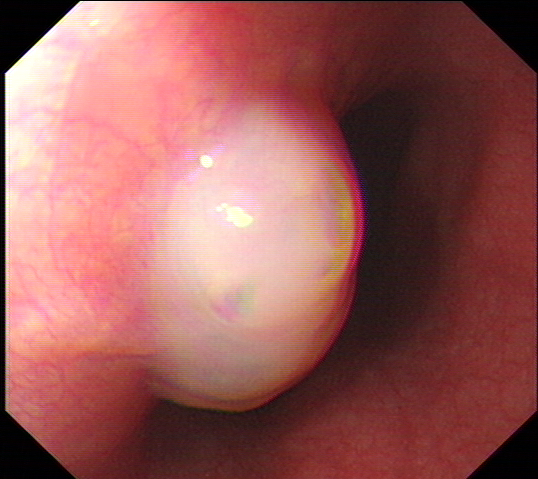

Supplement: Supplementary file 1 — 1. Plain scan CT. Plain scan CT of the esophagus showed the lesion located in the lower esophagus. The CT value is 16 Hu. 2. Enhanced CT. Enhanced CT of the esophagus showed the lesion located in the lower esophagus. The CT value is 38 Hu. 3. EUS. A large, whitish-yellow, translucent mass with a lustrous surface in the mid and distal esophagus, located 32 to 38 cm from the incisors. A honeycomb-like hypoechoic structure measuring 60 × 10 mm located in the submucosa with heterogenous echo pattern; the muscularis propria was intact. 4. EPMR. The esophageal mass was resected by endoscopic piecemeal mucosal resection (EPMR). 5. Pathology. Histology of the resected mass showing cystically dilated lymphatics in the surface squamous epithelium and in the submucosa. (hematoxylin and eosin [H&E], magnification ×200). [file 5747560.f1.zip › EPMR/EPMR/_0008.BMP]

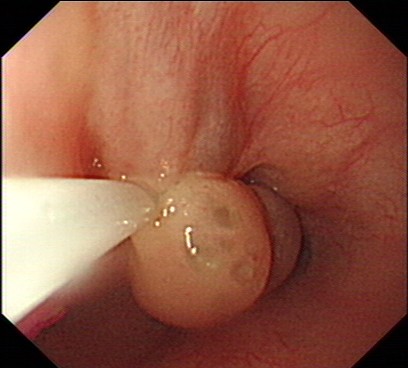

Supplement: Supplementary file 1 — 1. Plain scan CT. Plain scan CT of the esophagus showed the lesion located in the lower esophagus. The CT value is 16 Hu. 2. Enhanced CT. Enhanced CT of the esophagus showed the lesion located in the lower esophagus. The CT value is 38 Hu. 3. EUS. A large, whitish-yellow, translucent mass with a lustrous surface in the mid and distal esophagus, located 32 to 38 cm from the incisors. A honeycomb-like hypoechoic structure measuring 60 × 10 mm located in the submucosa with heterogenous echo pattern; the muscularis propria was intact. 4. EPMR. The esophageal mass was resected by endoscopic piecemeal mucosal resection (EPMR). 5. Pathology. Histology of the resected mass showing cystically dilated lymphatics in the surface squamous epithelium and in the submucosa. (hematoxylin and eosin [H&E], magnification ×200). [file 5747560.f1.zip › EPMR/EPMR/_0009.JPG]

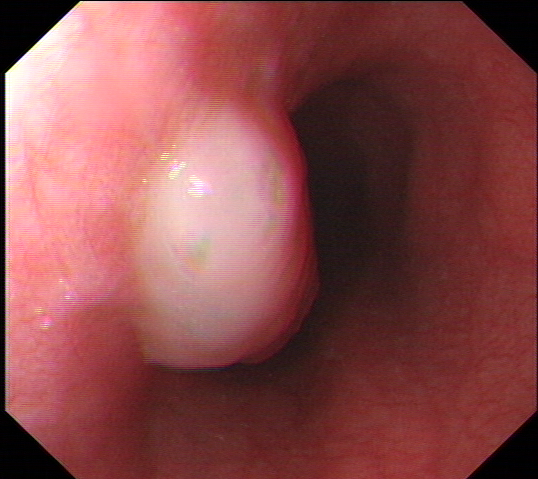

Supplement: Supplementary file 1 — 1. Plain scan CT. Plain scan CT of the esophagus showed the lesion located in the lower esophagus. The CT value is 16 Hu. 2. Enhanced CT. Enhanced CT of the esophagus showed the lesion located in the lower esophagus. The CT value is 38 Hu. 3. EUS. A large, whitish-yellow, translucent mass with a lustrous surface in the mid and distal esophagus, located 32 to 38 cm from the incisors. A honeycomb-like hypoechoic structure measuring 60 × 10 mm located in the submucosa with heterogenous echo pattern; the muscularis propria was intact. 4. EPMR. The esophageal mass was resected by endoscopic piecemeal mucosal resection (EPMR). 5. Pathology. Histology of the resected mass showing cystically dilated lymphatics in the surface squamous epithelium and in the submucosa. (hematoxylin and eosin [H&E], magnification ×200). [file 5747560.f1.zip › EPMR/EPMR/_0010.BMP]

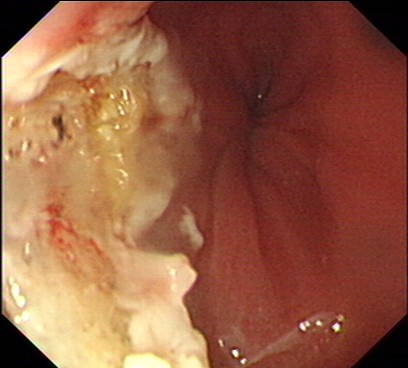

Supplement: Supplementary file 1 — 1. Plain scan CT. Plain scan CT of the esophagus showed the lesion located in the lower esophagus. The CT value is 16 Hu. 2. Enhanced CT. Enhanced CT of the esophagus showed the lesion located in the lower esophagus. The CT value is 38 Hu. 3. EUS. A large, whitish-yellow, translucent mass with a lustrous surface in the mid and distal esophagus, located 32 to 38 cm from the incisors. A honeycomb-like hypoechoic structure measuring 60 × 10 mm located in the submucosa with heterogenous echo pattern; the muscularis propria was intact. 4. EPMR. The esophageal mass was resected by endoscopic piecemeal mucosal resection (EPMR). 5. Pathology. Histology of the resected mass showing cystically dilated lymphatics in the surface squamous epithelium and in the submucosa. (hematoxylin and eosin [H&E], magnification ×200). [file 5747560.f1.zip › EPMR/EPMR/_0011.JPG]

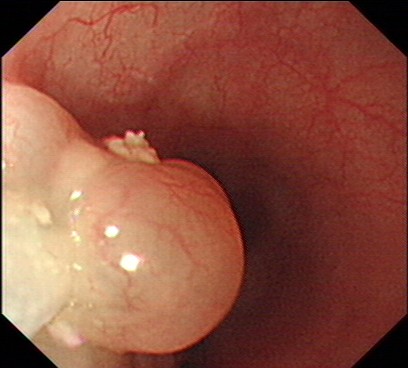

Supplement: Supplementary file 1 — 1. Plain scan CT. Plain scan CT of the esophagus showed the lesion located in the lower esophagus. The CT value is 16 Hu. 2. Enhanced CT. Enhanced CT of the esophagus showed the lesion located in the lower esophagus. The CT value is 38 Hu. 3. EUS. A large, whitish-yellow, translucent mass with a lustrous surface in the mid and distal esophagus, located 32 to 38 cm from the incisors. A honeycomb-like hypoechoic structure measuring 60 × 10 mm located in the submucosa with heterogenous echo pattern; the muscularis propria was intact. 4. EPMR. The esophageal mass was resected by endoscopic piecemeal mucosal resection (EPMR). 5. Pathology. Histology of the resected mass showing cystically dilated lymphatics in the surface squamous epithelium and in the submucosa. (hematoxylin and eosin [H&E], magnification ×200). [file 5747560.f1.zip › EPMR/EPMR/_0012.JPG]

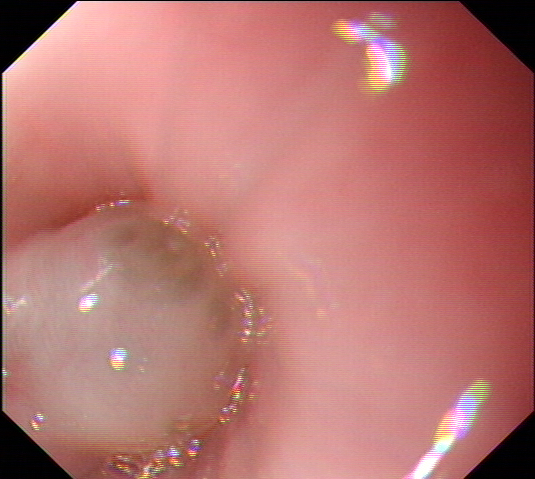

Supplement: Supplementary file 1 — 1. Plain scan CT. Plain scan CT of the esophagus showed the lesion located in the lower esophagus. The CT value is 16 Hu. 2. Enhanced CT. Enhanced CT of the esophagus showed the lesion located in the lower esophagus. The CT value is 38 Hu. 3. EUS. A large, whitish-yellow, translucent mass with a lustrous surface in the mid and distal esophagus, located 32 to 38 cm from the incisors. A honeycomb-like hypoechoic structure measuring 60 × 10 mm located in the submucosa with heterogenous echo pattern; the muscularis propria was intact. 4. EPMR. The esophageal mass was resected by endoscopic piecemeal mucosal resection (EPMR). 5. Pathology. Histology of the resected mass showing cystically dilated lymphatics in the surface squamous epithelium and in the submucosa. (hematoxylin and eosin [H&E], magnification ×200). [file 5747560.f1.zip › EPMR/EPMR/_0013.BMP]

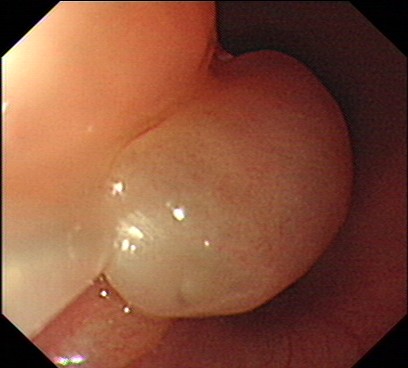

Supplement: Supplementary file 1 — 1. Plain scan CT. Plain scan CT of the esophagus showed the lesion located in the lower esophagus. The CT value is 16 Hu. 2. Enhanced CT. Enhanced CT of the esophagus showed the lesion located in the lower esophagus. The CT value is 38 Hu. 3. EUS. A large, whitish-yellow, translucent mass with a lustrous surface in the mid and distal esophagus, located 32 to 38 cm from the incisors. A honeycomb-like hypoechoic structure measuring 60 × 10 mm located in the submucosa with heterogenous echo pattern; the muscularis propria was intact. 4. EPMR. The esophageal mass was resected by endoscopic piecemeal mucosal resection (EPMR). 5. Pathology. Histology of the resected mass showing cystically dilated lymphatics in the surface squamous epithelium and in the submucosa. (hematoxylin and eosin [H&E], magnification ×200). [file 5747560.f1.zip › EPMR/EPMR/_0014.JPG]

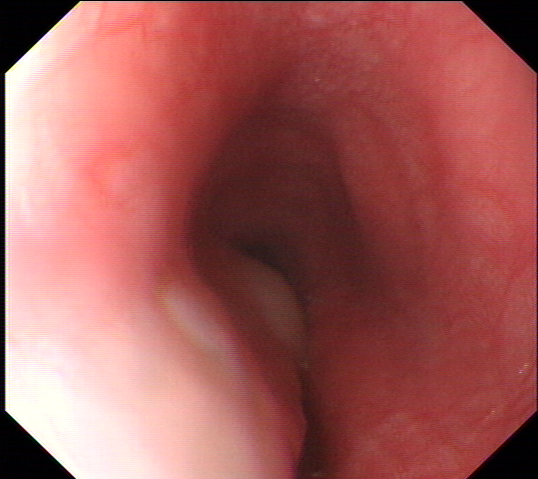

Supplement: Supplementary file 1 — 1. Plain scan CT. Plain scan CT of the esophagus showed the lesion located in the lower esophagus. The CT value is 16 Hu. 2. Enhanced CT. Enhanced CT of the esophagus showed the lesion located in the lower esophagus. The CT value is 38 Hu. 3. EUS. A large, whitish-yellow, translucent mass with a lustrous surface in the mid and distal esophagus, located 32 to 38 cm from the incisors. A honeycomb-like hypoechoic structure measuring 60 × 10 mm located in the submucosa with heterogenous echo pattern; the muscularis propria was intact. 4. EPMR. The esophageal mass was resected by endoscopic piecemeal mucosal resection (EPMR). 5. Pathology. Histology of the resected mass showing cystically dilated lymphatics in the surface squamous epithelium and in the submucosa. (hematoxylin and eosin [H&E], magnification ×200). [file 5747560.f1.zip › EPMR/EPMR/_0015.BMP]

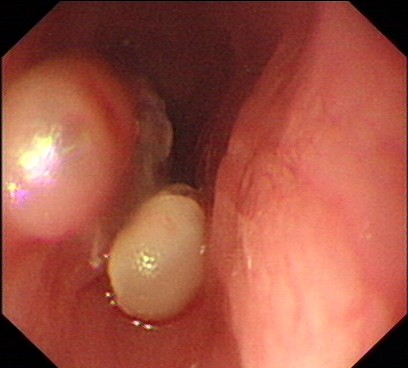

Supplement: Supplementary file 1 — 1. Plain scan CT. Plain scan CT of the esophagus showed the lesion located in the lower esophagus. The CT value is 16 Hu. 2. Enhanced CT. Enhanced CT of the esophagus showed the lesion located in the lower esophagus. The CT value is 38 Hu. 3. EUS. A large, whitish-yellow, translucent mass with a lustrous surface in the mid and distal esophagus, located 32 to 38 cm from the incisors. A honeycomb-like hypoechoic structure measuring 60 × 10 mm located in the submucosa with heterogenous echo pattern; the muscularis propria was intact. 4. EPMR. The esophageal mass was resected by endoscopic piecemeal mucosal resection (EPMR). 5. Pathology. Histology of the resected mass showing cystically dilated lymphatics in the surface squamous epithelium and in the submucosa. (hematoxylin and eosin [H&E], magnification ×200). [file 5747560.f1.zip › EPMR/EPMR/_0016.JPG]

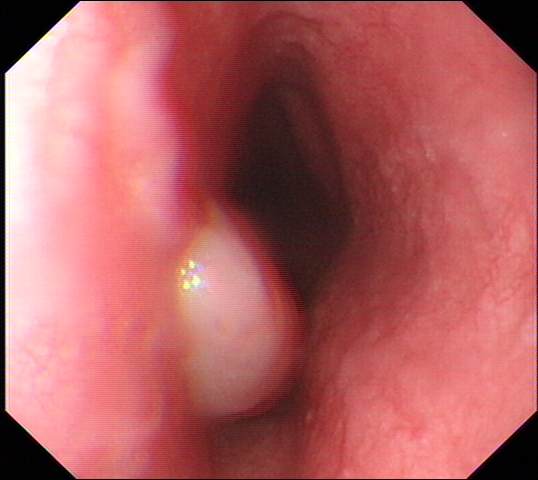

Supplement: Supplementary file 1 — 1. Plain scan CT. Plain scan CT of the esophagus showed the lesion located in the lower esophagus. The CT value is 16 Hu. 2. Enhanced CT. Enhanced CT of the esophagus showed the lesion located in the lower esophagus. The CT value is 38 Hu. 3. EUS. A large, whitish-yellow, translucent mass with a lustrous surface in the mid and distal esophagus, located 32 to 38 cm from the incisors. A honeycomb-like hypoechoic structure measuring 60 × 10 mm located in the submucosa with heterogenous echo pattern; the muscularis propria was intact. 4. EPMR. The esophageal mass was resected by endoscopic piecemeal mucosal resection (EPMR). 5. Pathology. Histology of the resected mass showing cystically dilated lymphatics in the surface squamous epithelium and in the submucosa. (hematoxylin and eosin [H&E], magnification ×200). [file 5747560.f1.zip › EPMR/EPMR/_0017.BMP]

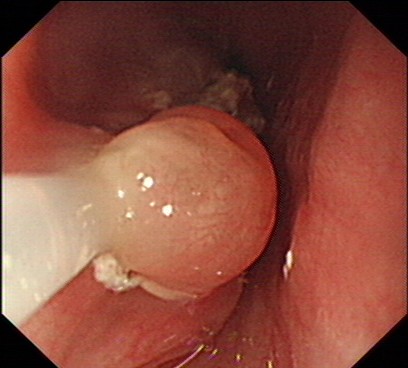

Supplement: Supplementary file 1 — 1. Plain scan CT. Plain scan CT of the esophagus showed the lesion located in the lower esophagus. The CT value is 16 Hu. 2. Enhanced CT. Enhanced CT of the esophagus showed the lesion located in the lower esophagus. The CT value is 38 Hu. 3. EUS. A large, whitish-yellow, translucent mass with a lustrous surface in the mid and distal esophagus, located 32 to 38 cm from the incisors. A honeycomb-like hypoechoic structure measuring 60 × 10 mm located in the submucosa with heterogenous echo pattern; the muscularis propria was intact. 4. EPMR. The esophageal mass was resected by endoscopic piecemeal mucosal resection (EPMR). 5. Pathology. Histology of the resected mass showing cystically dilated lymphatics in the surface squamous epithelium and in the submucosa. (hematoxylin and eosin [H&E], magnification ×200). [file 5747560.f1.zip › EPMR/EPMR/_0018.JPG]

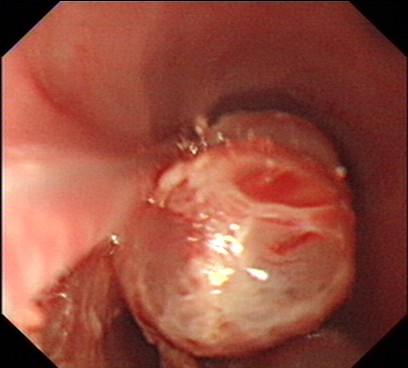

Supplement: Supplementary file 1 — 1. Plain scan CT. Plain scan CT of the esophagus showed the lesion located in the lower esophagus. The CT value is 16 Hu. 2. Enhanced CT. Enhanced CT of the esophagus showed the lesion located in the lower esophagus. The CT value is 38 Hu. 3. EUS. A large, whitish-yellow, translucent mass with a lustrous surface in the mid and distal esophagus, located 32 to 38 cm from the incisors. A honeycomb-like hypoechoic structure measuring 60 × 10 mm located in the submucosa with heterogenous echo pattern; the muscularis propria was intact. 4. EPMR. The esophageal mass was resected by endoscopic piecemeal mucosal resection (EPMR). 5. Pathology. Histology of the resected mass showing cystically dilated lymphatics in the surface squamous epithelium and in the submucosa. (hematoxylin and eosin [H&E], magnification ×200). [file 5747560.f1.zip › EPMR/EPMR/_0019.JPG]

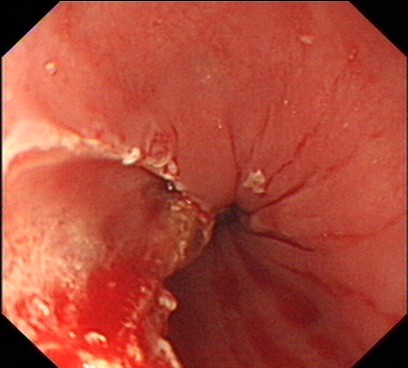

Supplement: Supplementary file 1 — 1. Plain scan CT. Plain scan CT of the esophagus showed the lesion located in the lower esophagus. The CT value is 16 Hu. 2. Enhanced CT. Enhanced CT of the esophagus showed the lesion located in the lower esophagus. The CT value is 38 Hu. 3. EUS. A large, whitish-yellow, translucent mass with a lustrous surface in the mid and distal esophagus, located 32 to 38 cm from the incisors. A honeycomb-like hypoechoic structure measuring 60 × 10 mm located in the submucosa with heterogenous echo pattern; the muscularis propria was intact. 4. EPMR. The esophageal mass was resected by endoscopic piecemeal mucosal resection (EPMR). 5. Pathology. Histology of the resected mass showing cystically dilated lymphatics in the surface squamous epithelium and in the submucosa. (hematoxylin and eosin [H&E], magnification ×200). [file 5747560.f1.zip › EPMR/EPMR/_0020.JPG]

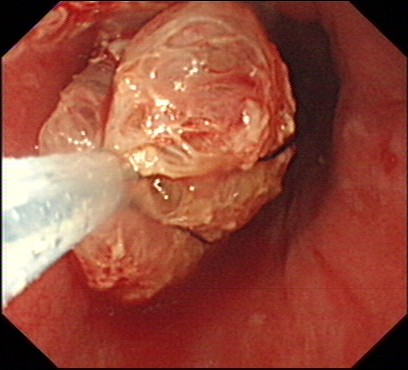

Supplement: Supplementary file 1 — 1. Plain scan CT. Plain scan CT of the esophagus showed the lesion located in the lower esophagus. The CT value is 16 Hu. 2. Enhanced CT. Enhanced CT of the esophagus showed the lesion located in the lower esophagus. The CT value is 38 Hu. 3. EUS. A large, whitish-yellow, translucent mass with a lustrous surface in the mid and distal esophagus, located 32 to 38 cm from the incisors. A honeycomb-like hypoechoic structure measuring 60 × 10 mm located in the submucosa with heterogenous echo pattern; the muscularis propria was intact. 4. EPMR. The esophageal mass was resected by endoscopic piecemeal mucosal resection (EPMR). 5. Pathology. Histology of the resected mass showing cystically dilated lymphatics in the surface squamous epithelium and in the submucosa. (hematoxylin and eosin [H&E], magnification ×200). [file 5747560.f1.zip › EPMR/EPMR/_0021.JPG]

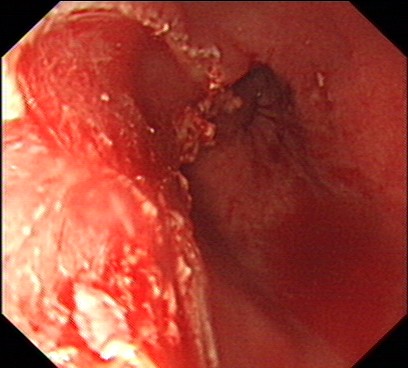

Supplement: Supplementary file 1 — 1. Plain scan CT. Plain scan CT of the esophagus showed the lesion located in the lower esophagus. The CT value is 16 Hu. 2. Enhanced CT. Enhanced CT of the esophagus showed the lesion located in the lower esophagus. The CT value is 38 Hu. 3. EUS. A large, whitish-yellow, translucent mass with a lustrous surface in the mid and distal esophagus, located 32 to 38 cm from the incisors. A honeycomb-like hypoechoic structure measuring 60 × 10 mm located in the submucosa with heterogenous echo pattern; the muscularis propria was intact. 4. EPMR. The esophageal mass was resected by endoscopic piecemeal mucosal resection (EPMR). 5. Pathology. Histology of the resected mass showing cystically dilated lymphatics in the surface squamous epithelium and in the submucosa. (hematoxylin and eosin [H&E], magnification ×200). [file 5747560.f1.zip › EPMR/EPMR/_0022.JPG]

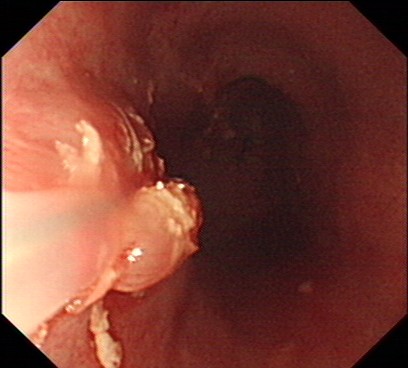

Supplement: Supplementary file 1 — 1. Plain scan CT. Plain scan CT of the esophagus showed the lesion located in the lower esophagus. The CT value is 16 Hu. 2. Enhanced CT. Enhanced CT of the esophagus showed the lesion located in the lower esophagus. The CT value is 38 Hu. 3. EUS. A large, whitish-yellow, translucent mass with a lustrous surface in the mid and distal esophagus, located 32 to 38 cm from the incisors. A honeycomb-like hypoechoic structure measuring 60 × 10 mm located in the submucosa with heterogenous echo pattern; the muscularis propria was intact. 4. EPMR. The esophageal mass was resected by endoscopic piecemeal mucosal resection (EPMR). 5. Pathology. Histology of the resected mass showing cystically dilated lymphatics in the surface squamous epithelium and in the submucosa. (hematoxylin and eosin [H&E], magnification ×200). [file 5747560.f1.zip › EPMR/EPMR/_0023.JPG]

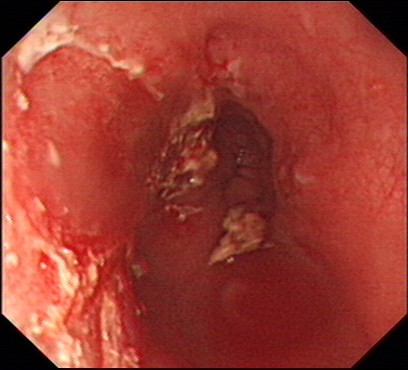

Supplement: Supplementary file 1 — 1. Plain scan CT. Plain scan CT of the esophagus showed the lesion located in the lower esophagus. The CT value is 16 Hu. 2. Enhanced CT. Enhanced CT of the esophagus showed the lesion located in the lower esophagus. The CT value is 38 Hu. 3. EUS. A large, whitish-yellow, translucent mass with a lustrous surface in the mid and distal esophagus, located 32 to 38 cm from the incisors. A honeycomb-like hypoechoic structure measuring 60 × 10 mm located in the submucosa with heterogenous echo pattern; the muscularis propria was intact. 4. EPMR. The esophageal mass was resected by endoscopic piecemeal mucosal resection (EPMR). 5. Pathology. Histology of the resected mass showing cystically dilated lymphatics in the surface squamous epithelium and in the submucosa. (hematoxylin and eosin [H&E], magnification ×200). [file 5747560.f1.zip › EPMR/EPMR/_0024.JPG]

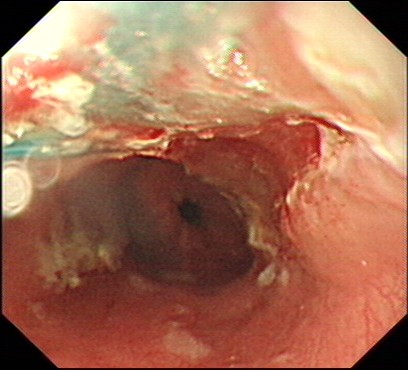

Supplement: Supplementary file 1 — 1. Plain scan CT. Plain scan CT of the esophagus showed the lesion located in the lower esophagus. The CT value is 16 Hu. 2. Enhanced CT. Enhanced CT of the esophagus showed the lesion located in the lower esophagus. The CT value is 38 Hu. 3. EUS. A large, whitish-yellow, translucent mass with a lustrous surface in the mid and distal esophagus, located 32 to 38 cm from the incisors. A honeycomb-like hypoechoic structure measuring 60 × 10 mm located in the submucosa with heterogenous echo pattern; the muscularis propria was intact. 4. EPMR. The esophageal mass was resected by endoscopic piecemeal mucosal resection (EPMR). 5. Pathology. Histology of the resected mass showing cystically dilated lymphatics in the surface squamous epithelium and in the submucosa. (hematoxylin and eosin [H&E], magnification ×200). [file 5747560.f1.zip › EPMR/EPMR/_0025.JPG]

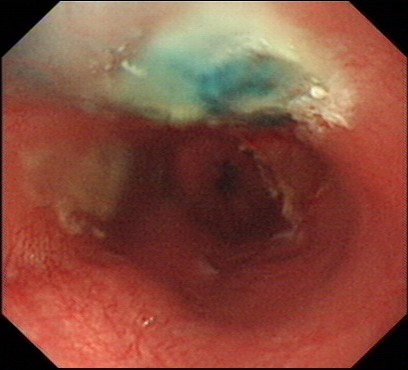

Supplement: Supplementary file 1 — 1. Plain scan CT. Plain scan CT of the esophagus showed the lesion located in the lower esophagus. The CT value is 16 Hu. 2. Enhanced CT. Enhanced CT of the esophagus showed the lesion located in the lower esophagus. The CT value is 38 Hu. 3. EUS. A large, whitish-yellow, translucent mass with a lustrous surface in the mid and distal esophagus, located 32 to 38 cm from the incisors. A honeycomb-like hypoechoic structure measuring 60 × 10 mm located in the submucosa with heterogenous echo pattern; the muscularis propria was intact. 4. EPMR. The esophageal mass was resected by endoscopic piecemeal mucosal resection (EPMR). 5. Pathology. Histology of the resected mass showing cystically dilated lymphatics in the surface squamous epithelium and in the submucosa. (hematoxylin and eosin [H&E], magnification ×200). [file 5747560.f1.zip › EPMR/EPMR/_0026.JPG]

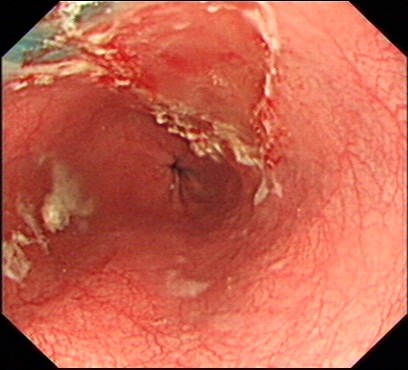

Supplement: Supplementary file 1 — 1. Plain scan CT. Plain scan CT of the esophagus showed the lesion located in the lower esophagus. The CT value is 16 Hu. 2. Enhanced CT. Enhanced CT of the esophagus showed the lesion located in the lower esophagus. The CT value is 38 Hu. 3. EUS. A large, whitish-yellow, translucent mass with a lustrous surface in the mid and distal esophagus, located 32 to 38 cm from the incisors. A honeycomb-like hypoechoic structure measuring 60 × 10 mm located in the submucosa with heterogenous echo pattern; the muscularis propria was intact. 4. EPMR. The esophageal mass was resected by endoscopic piecemeal mucosal resection (EPMR). 5. Pathology. Histology of the resected mass showing cystically dilated lymphatics in the surface squamous epithelium and in the submucosa. (hematoxylin and eosin [H&E], magnification ×200). [file 5747560.f1.zip › EPMR/EPMR/_0027.JPG]

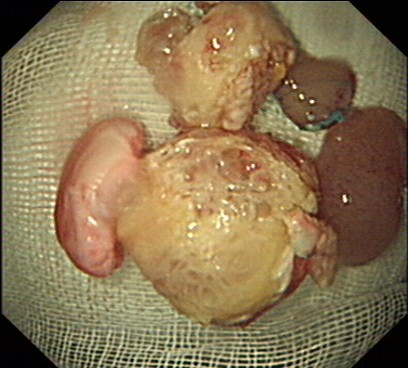

Supplement: Supplementary file 1 — 1. Plain scan CT. Plain scan CT of the esophagus showed the lesion located in the lower esophagus. The CT value is 16 Hu. 2. Enhanced CT. Enhanced CT of the esophagus showed the lesion located in the lower esophagus. The CT value is 38 Hu. 3. EUS. A large, whitish-yellow, translucent mass with a lustrous surface in the mid and distal esophagus, located 32 to 38 cm from the incisors. A honeycomb-like hypoechoic structure measuring 60 × 10 mm located in the submucosa with heterogenous echo pattern; the muscularis propria was intact. 4. EPMR. The esophageal mass was resected by endoscopic piecemeal mucosal resection (EPMR). 5. Pathology. Histology of the resected mass showing cystically dilated lymphatics in the surface squamous epithelium and in the submucosa. (hematoxylin and eosin [H&E], magnification ×200). [file 5747560.f1.zip › EPMR/EPMR/_0028.JPG]

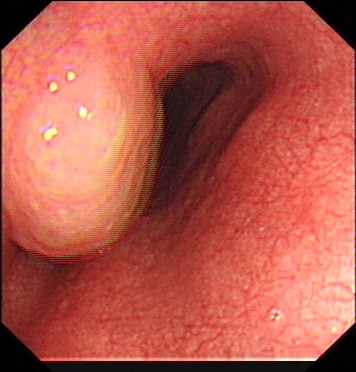

Supplement: Supplementary file 1 — 1. Plain scan CT. Plain scan CT of the esophagus showed the lesion located in the lower esophagus. The CT value is 16 Hu. 2. Enhanced CT. Enhanced CT of the esophagus showed the lesion located in the lower esophagus. The CT value is 38 Hu. 3. EUS. A large, whitish-yellow, translucent mass with a lustrous surface in the mid and distal esophagus, located 32 to 38 cm from the incisors. A honeycomb-like hypoechoic structure measuring 60 × 10 mm located in the submucosa with heterogenous echo pattern; the muscularis propria was intact. 4. EPMR. The esophageal mass was resected by endoscopic piecemeal mucosal resection (EPMR). 5. Pathology. Histology of the resected mass showing cystically dilated lymphatics in the surface squamous epithelium and in the submucosa. (hematoxylin and eosin [H&E], magnification ×200). [file 5747560.f1.zip › EPMR/EUS/_0001.JPG]

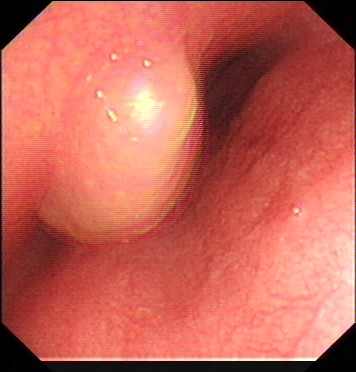

Supplement: Supplementary file 1 — 1. Plain scan CT. Plain scan CT of the esophagus showed the lesion located in the lower esophagus. The CT value is 16 Hu. 2. Enhanced CT. Enhanced CT of the esophagus showed the lesion located in the lower esophagus. The CT value is 38 Hu. 3. EUS. A large, whitish-yellow, translucent mass with a lustrous surface in the mid and distal esophagus, located 32 to 38 cm from the incisors. A honeycomb-like hypoechoic structure measuring 60 × 10 mm located in the submucosa with heterogenous echo pattern; the muscularis propria was intact. 4. EPMR. The esophageal mass was resected by endoscopic piecemeal mucosal resection (EPMR). 5. Pathology. Histology of the resected mass showing cystically dilated lymphatics in the surface squamous epithelium and in the submucosa. (hematoxylin and eosin [H&E], magnification ×200). [file 5747560.f1.zip › EPMR/EUS/_0002.JPG]

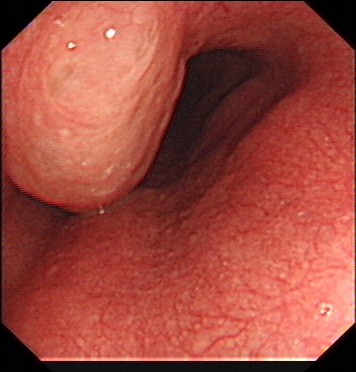

Supplement: Supplementary file 1 — 1. Plain scan CT. Plain scan CT of the esophagus showed the lesion located in the lower esophagus. The CT value is 16 Hu. 2. Enhanced CT. Enhanced CT of the esophagus showed the lesion located in the lower esophagus. The CT value is 38 Hu. 3. EUS. A large, whitish-yellow, translucent mass with a lustrous surface in the mid and distal esophagus, located 32 to 38 cm from the incisors. A honeycomb-like hypoechoic structure measuring 60 × 10 mm located in the submucosa with heterogenous echo pattern; the muscularis propria was intact. 4. EPMR. The esophageal mass was resected by endoscopic piecemeal mucosal resection (EPMR). 5. Pathology. Histology of the resected mass showing cystically dilated lymphatics in the surface squamous epithelium and in the submucosa. (hematoxylin and eosin [H&E], magnification ×200). [file 5747560.f1.zip › EPMR/EUS/_0003.JPG]

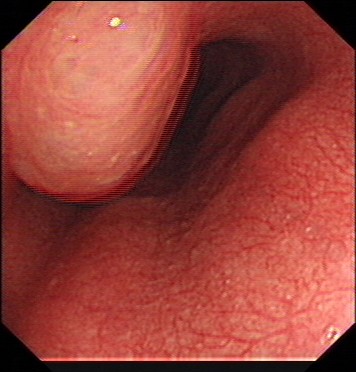

Supplement: Supplementary file 1 — 1. Plain scan CT. Plain scan CT of the esophagus showed the lesion located in the lower esophagus. The CT value is 16 Hu. 2. Enhanced CT. Enhanced CT of the esophagus showed the lesion located in the lower esophagus. The CT value is 38 Hu. 3. EUS. A large, whitish-yellow, translucent mass with a lustrous surface in the mid and distal esophagus, located 32 to 38 cm from the incisors. A honeycomb-like hypoechoic structure measuring 60 × 10 mm located in the submucosa with heterogenous echo pattern; the muscularis propria was intact. 4. EPMR. The esophageal mass was resected by endoscopic piecemeal mucosal resection (EPMR). 5. Pathology. Histology of the resected mass showing cystically dilated lymphatics in the surface squamous epithelium and in the submucosa. (hematoxylin and eosin [H&E], magnification ×200). [file 5747560.f1.zip › EPMR/EUS/_0004.JPG]

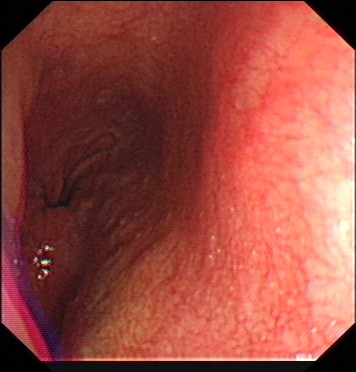

Supplement: Supplementary file 1 — 1. Plain scan CT. Plain scan CT of the esophagus showed the lesion located in the lower esophagus. The CT value is 16 Hu. 2. Enhanced CT. Enhanced CT of the esophagus showed the lesion located in the lower esophagus. The CT value is 38 Hu. 3. EUS. A large, whitish-yellow, translucent mass with a lustrous surface in the mid and distal esophagus, located 32 to 38 cm from the incisors. A honeycomb-like hypoechoic structure measuring 60 × 10 mm located in the submucosa with heterogenous echo pattern; the muscularis propria was intact. 4. EPMR. The esophageal mass was resected by endoscopic piecemeal mucosal resection (EPMR). 5. Pathology. Histology of the resected mass showing cystically dilated lymphatics in the surface squamous epithelium and in the submucosa. (hematoxylin and eosin [H&E], magnification ×200). [file 5747560.f1.zip › EPMR/EUS/_0005.JPG]

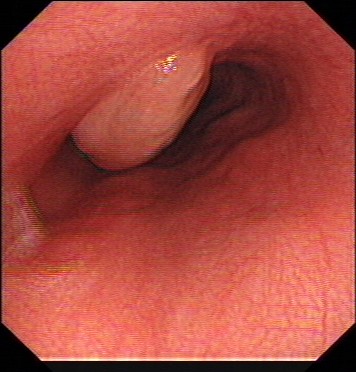

Supplement: Supplementary file 1 — 1. Plain scan CT. Plain scan CT of the esophagus showed the lesion located in the lower esophagus. The CT value is 16 Hu. 2. Enhanced CT. Enhanced CT of the esophagus showed the lesion located in the lower esophagus. The CT value is 38 Hu. 3. EUS. A large, whitish-yellow, translucent mass with a lustrous surface in the mid and distal esophagus, located 32 to 38 cm from the incisors. A honeycomb-like hypoechoic structure measuring 60 × 10 mm located in the submucosa with heterogenous echo pattern; the muscularis propria was intact. 4. EPMR. The esophageal mass was resected by endoscopic piecemeal mucosal resection (EPMR). 5. Pathology. Histology of the resected mass showing cystically dilated lymphatics in the surface squamous epithelium and in the submucosa. (hematoxylin and eosin [H&E], magnification ×200). [file 5747560.f1.zip › EPMR/EUS/_0006.JPG]

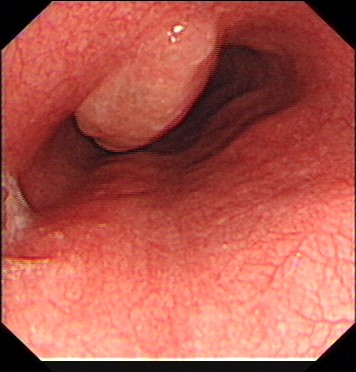

Supplement: Supplementary file 1 — 1. Plain scan CT. Plain scan CT of the esophagus showed the lesion located in the lower esophagus. The CT value is 16 Hu. 2. Enhanced CT. Enhanced CT of the esophagus showed the lesion located in the lower esophagus. The CT value is 38 Hu. 3. EUS. A large, whitish-yellow, translucent mass with a lustrous surface in the mid and distal esophagus, located 32 to 38 cm from the incisors. A honeycomb-like hypoechoic structure measuring 60 × 10 mm located in the submucosa with heterogenous echo pattern; the muscularis propria was intact. 4. EPMR. The esophageal mass was resected by endoscopic piecemeal mucosal resection (EPMR). 5. Pathology. Histology of the resected mass showing cystically dilated lymphatics in the surface squamous epithelium and in the submucosa. (hematoxylin and eosin [H&E], magnification ×200). [file 5747560.f1.zip › EPMR/EUS/_0007.JPG]

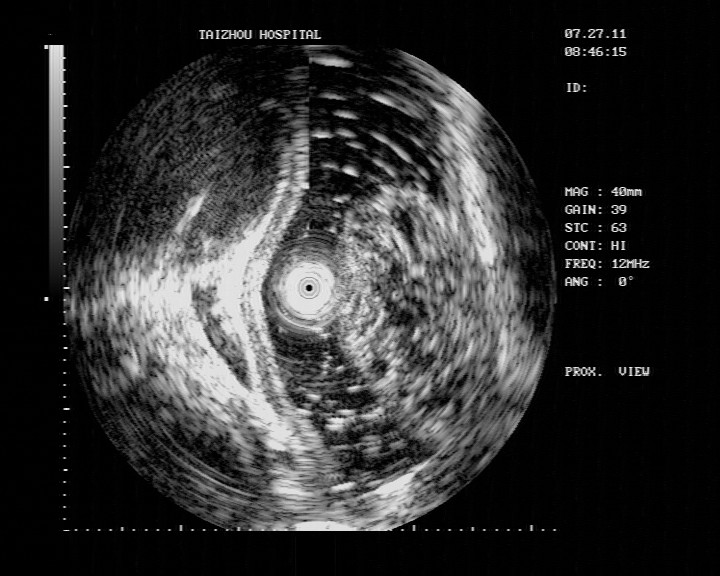

Supplement: Supplementary file 1 — 1. Plain scan CT. Plain scan CT of the esophagus showed the lesion located in the lower esophagus. The CT value is 16 Hu. 2. Enhanced CT. Enhanced CT of the esophagus showed the lesion located in the lower esophagus. The CT value is 38 Hu. 3. EUS. A large, whitish-yellow, translucent mass with a lustrous surface in the mid and distal esophagus, located 32 to 38 cm from the incisors. A honeycomb-like hypoechoic structure measuring 60 × 10 mm located in the submucosa with heterogenous echo pattern; the muscularis propria was intact. 4. EPMR. The esophageal mass was resected by endoscopic piecemeal mucosal resection (EPMR). 5. Pathology. Histology of the resected mass showing cystically dilated lymphatics in the surface squamous epithelium and in the submucosa. (hematoxylin and eosin [H&E], magnification ×200). [file 5747560.f1.zip › EPMR/EUS/_0008.JPG]

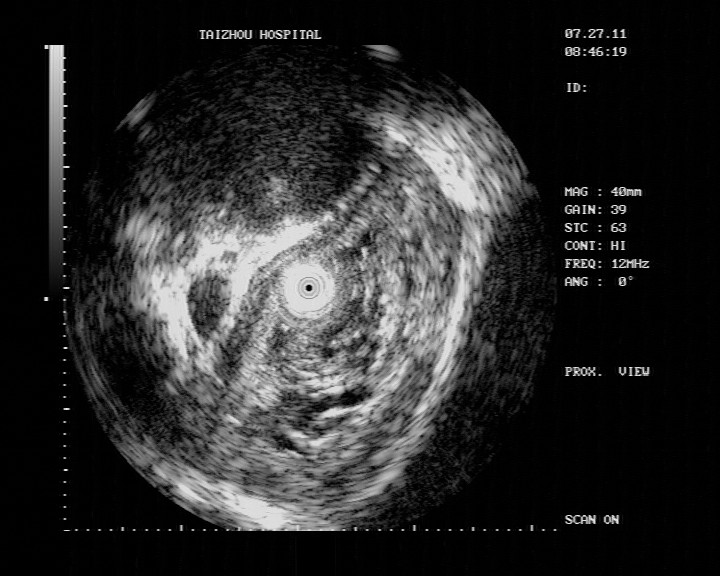

Supplement: Supplementary file 1 — 1. Plain scan CT. Plain scan CT of the esophagus showed the lesion located in the lower esophagus. The CT value is 16 Hu. 2. Enhanced CT. Enhanced CT of the esophagus showed the lesion located in the lower esophagus. The CT value is 38 Hu. 3. EUS. A large, whitish-yellow, translucent mass with a lustrous surface in the mid and distal esophagus, located 32 to 38 cm from the incisors. A honeycomb-like hypoechoic structure measuring 60 × 10 mm located in the submucosa with heterogenous echo pattern; the muscularis propria was intact. 4. EPMR. The esophageal mass was resected by endoscopic piecemeal mucosal resection (EPMR). 5. Pathology. Histology of the resected mass showing cystically dilated lymphatics in the surface squamous epithelium and in the submucosa. (hematoxylin and eosin [H&E], magnification ×200). [file 5747560.f1.zip › EPMR/EUS/_0009.JPG]

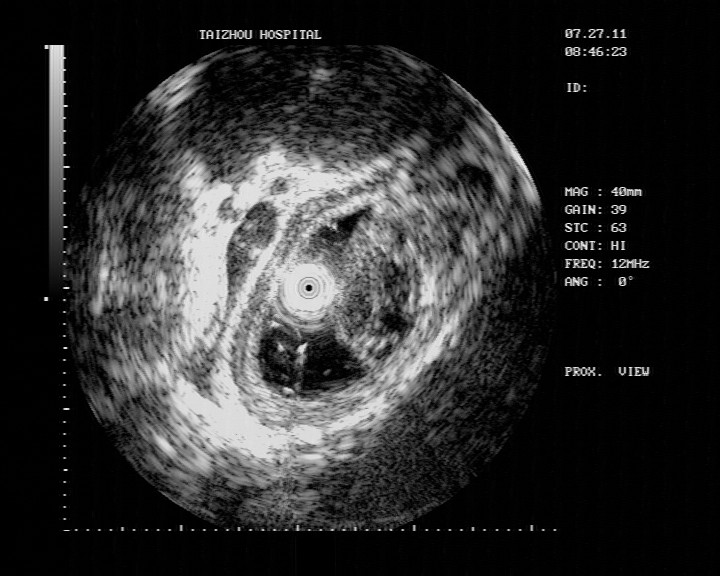

Supplement: Supplementary file 1 — 1. Plain scan CT. Plain scan CT of the esophagus showed the lesion located in the lower esophagus. The CT value is 16 Hu. 2. Enhanced CT. Enhanced CT of the esophagus showed the lesion located in the lower esophagus. The CT value is 38 Hu. 3. EUS. A large, whitish-yellow, translucent mass with a lustrous surface in the mid and distal esophagus, located 32 to 38 cm from the incisors. A honeycomb-like hypoechoic structure measuring 60 × 10 mm located in the submucosa with heterogenous echo pattern; the muscularis propria was intact. 4. EPMR. The esophageal mass was resected by endoscopic piecemeal mucosal resection (EPMR). 5. Pathology. Histology of the resected mass showing cystically dilated lymphatics in the surface squamous epithelium and in the submucosa. (hematoxylin and eosin [H&E], magnification ×200). [file 5747560.f1.zip › EPMR/EUS/_0010.JPG]

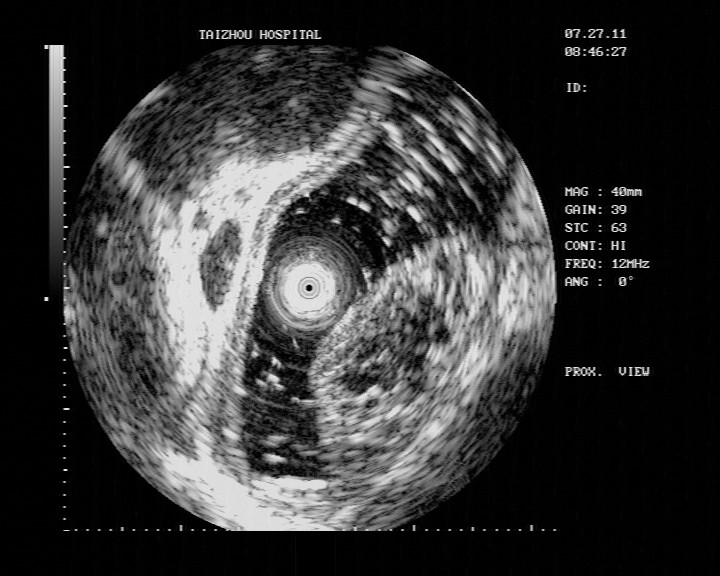

Supplement: Supplementary file 1 — 1. Plain scan CT. Plain scan CT of the esophagus showed the lesion located in the lower esophagus. The CT value is 16 Hu. 2. Enhanced CT. Enhanced CT of the esophagus showed the lesion located in the lower esophagus. The CT value is 38 Hu. 3. EUS. A large, whitish-yellow, translucent mass with a lustrous surface in the mid and distal esophagus, located 32 to 38 cm from the incisors. A honeycomb-like hypoechoic structure measuring 60 × 10 mm located in the submucosa with heterogenous echo pattern; the muscularis propria was intact. 4. EPMR. The esophageal mass was resected by endoscopic piecemeal mucosal resection (EPMR). 5. Pathology. Histology of the resected mass showing cystically dilated lymphatics in the surface squamous epithelium and in the submucosa. (hematoxylin and eosin [H&E], magnification ×200). [file 5747560.f1.zip › EPMR/EUS/_0011.JPG]

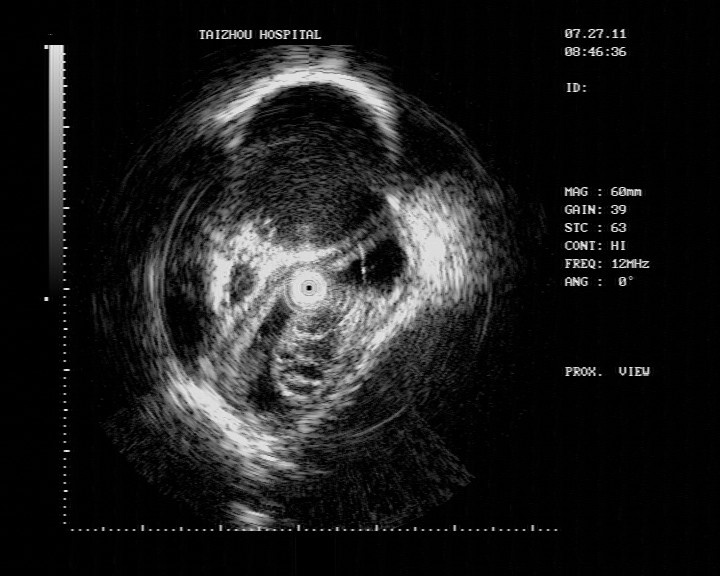

Supplement: Supplementary file 1 — 1. Plain scan CT. Plain scan CT of the esophagus showed the lesion located in the lower esophagus. The CT value is 16 Hu. 2. Enhanced CT. Enhanced CT of the esophagus showed the lesion located in the lower esophagus. The CT value is 38 Hu. 3. EUS. A large, whitish-yellow, translucent mass with a lustrous surface in the mid and distal esophagus, located 32 to 38 cm from the incisors. A honeycomb-like hypoechoic structure measuring 60 × 10 mm located in the submucosa with heterogenous echo pattern; the muscularis propria was intact. 4. EPMR. The esophageal mass was resected by endoscopic piecemeal mucosal resection (EPMR). 5. Pathology. Histology of the resected mass showing cystically dilated lymphatics in the surface squamous epithelium and in the submucosa. (hematoxylin and eosin [H&E], magnification ×200). [file 5747560.f1.zip › EPMR/EUS/_0012.JPG]

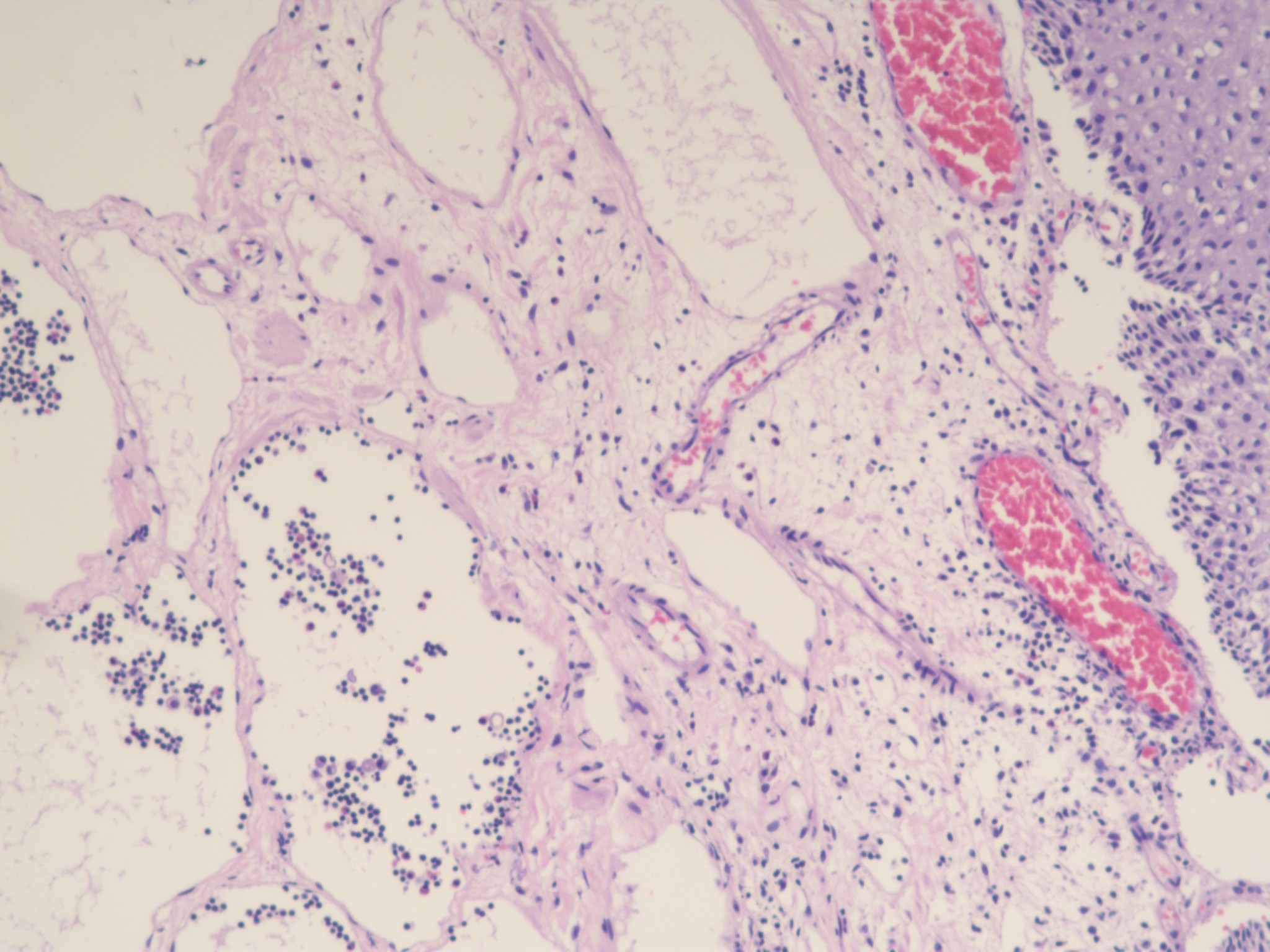

Supplement: Supplementary file 1 — 1. Plain scan CT. Plain scan CT of the esophagus showed the lesion located in the lower esophagus. The CT value is 16 Hu. 2. Enhanced CT. Enhanced CT of the esophagus showed the lesion located in the lower esophagus. The CT value is 38 Hu. 3. EUS. A large, whitish-yellow, translucent mass with a lustrous surface in the mid and distal esophagus, located 32 to 38 cm from the incisors. A honeycomb-like hypoechoic structure measuring 60 × 10 mm located in the submucosa with heterogenous echo pattern; the muscularis propria was intact. 4. EPMR. The esophageal mass was resected by endoscopic piecemeal mucosal resection (EPMR). 5. Pathology. Histology of the resected mass showing cystically dilated lymphatics in the surface squamous epithelium and in the submucosa. (hematoxylin and eosin [H&E], magnification ×200). [file 5747560.f1.zip › EPMR/Pathology/_0X10.JPG]

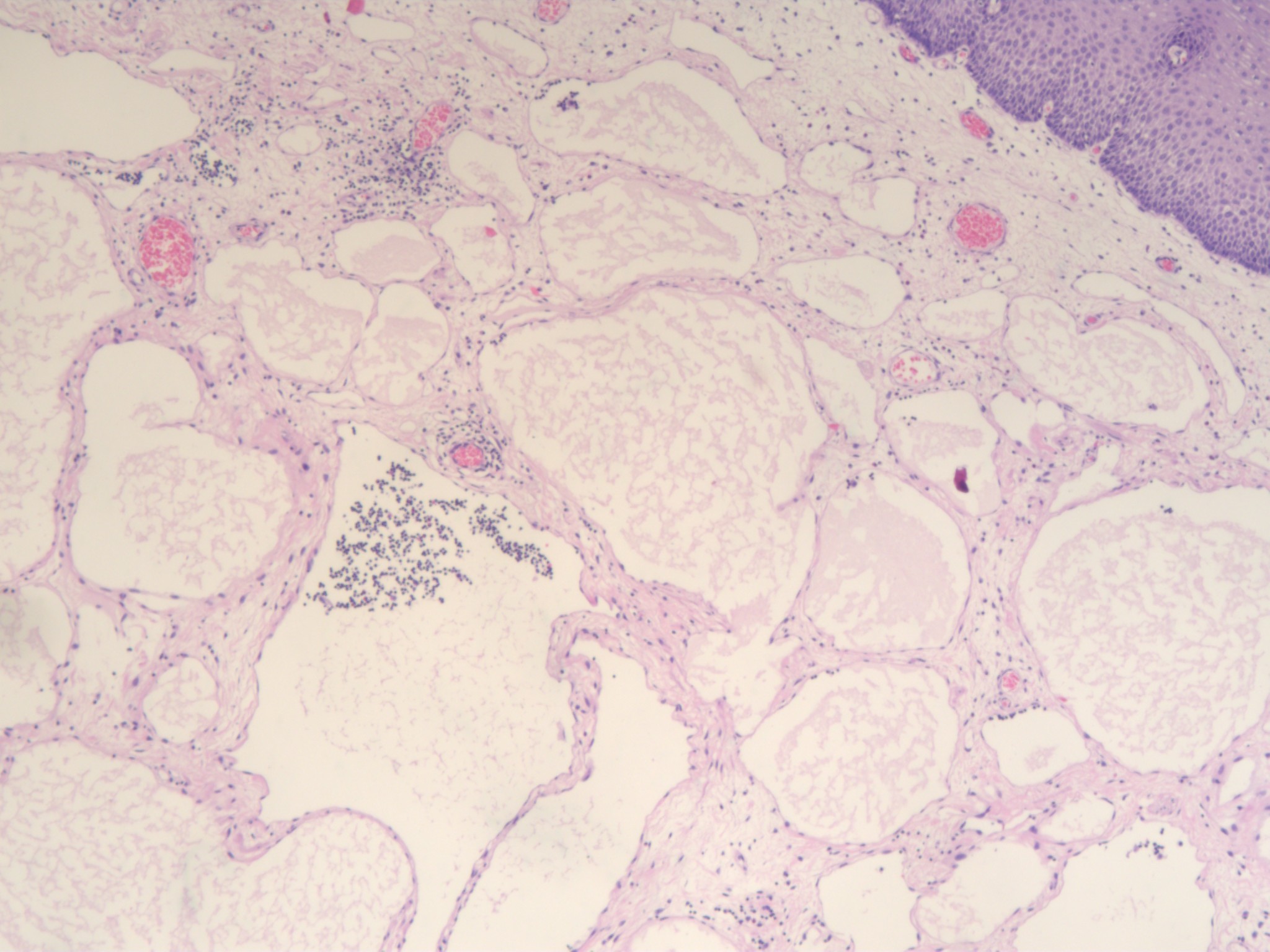

Supplement: Supplementary file 1 — 1. Plain scan CT. Plain scan CT of the esophagus showed the lesion located in the lower esophagus. The CT value is 16 Hu. 2. Enhanced CT. Enhanced CT of the esophagus showed the lesion located in the lower esophagus. The CT value is 38 Hu. 3. EUS. A large, whitish-yellow, translucent mass with a lustrous surface in the mid and distal esophagus, located 32 to 38 cm from the incisors. A honeycomb-like hypoechoic structure measuring 60 × 10 mm located in the submucosa with heterogenous echo pattern; the muscularis propria was intact. 4. EPMR. The esophageal mass was resected by endoscopic piecemeal mucosal resection (EPMR). 5. Pathology. Histology of the resected mass showing cystically dilated lymphatics in the surface squamous epithelium and in the submucosa. (hematoxylin and eosin [H&E], magnification ×200). [file 5747560.f1.zip › EPMR/Pathology/_X10.JPG]

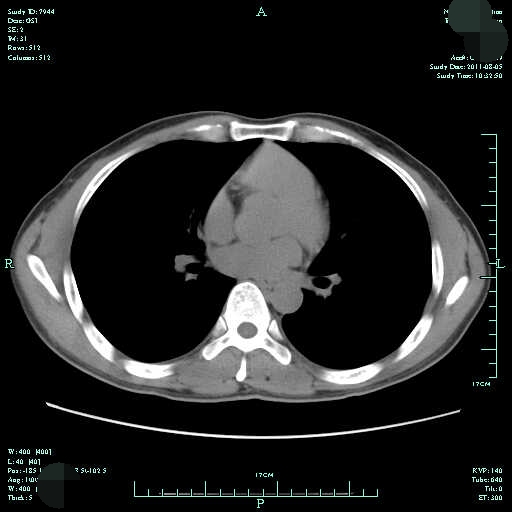

Supplement: Supplementary file 1 — 1. Plain scan CT. Plain scan CT of the esophagus showed the lesion located in the lower esophagus. The CT value is 16 Hu. 2. Enhanced CT. Enhanced CT of the esophagus showed the lesion located in the lower esophagus. The CT value is 38 Hu. 3. EUS. A large, whitish-yellow, translucent mass with a lustrous surface in the mid and distal esophagus, located 32 to 38 cm from the incisors. A honeycomb-like hypoechoic structure measuring 60 × 10 mm located in the submucosa with heterogenous echo pattern; the muscularis propria was intact. 4. EPMR. The esophageal mass was resected by endoscopic piecemeal mucosal resection (EPMR). 5. Pathology. Histology of the resected mass showing cystically dilated lymphatics in the surface squamous epithelium and in the submucosa. (hematoxylin and eosin [H&E], magnification ×200). [file 5747560.f1.zip › EPMR/Plain scan CT/exported0030_看图王.jpg]

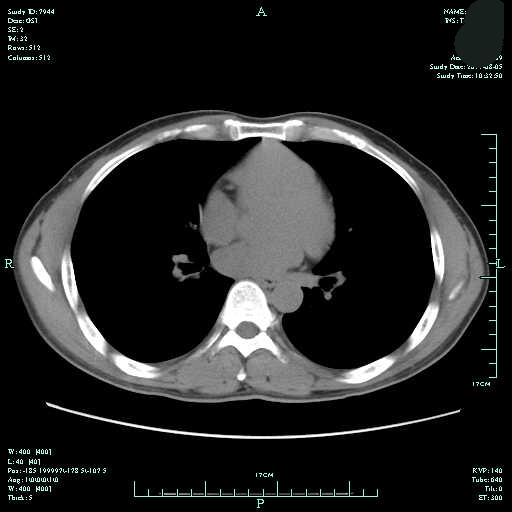

Supplement: Supplementary file 1 — 1. Plain scan CT. Plain scan CT of the esophagus showed the lesion located in the lower esophagus. The CT value is 16 Hu. 2. Enhanced CT. Enhanced CT of the esophagus showed the lesion located in the lower esophagus. The CT value is 38 Hu. 3. EUS. A large, whitish-yellow, translucent mass with a lustrous surface in the mid and distal esophagus, located 32 to 38 cm from the incisors. A honeycomb-like hypoechoic structure measuring 60 × 10 mm located in the submucosa with heterogenous echo pattern; the muscularis propria was intact. 4. EPMR. The esophageal mass was resected by endoscopic piecemeal mucosal resection (EPMR). 5. Pathology. Histology of the resected mass showing cystically dilated lymphatics in the surface squamous epithelium and in the submucosa. (hematoxylin and eosin [H&E], magnification ×200). [file 5747560.f1.zip › EPMR/Plain scan CT/exported0031_看图王.jpg]

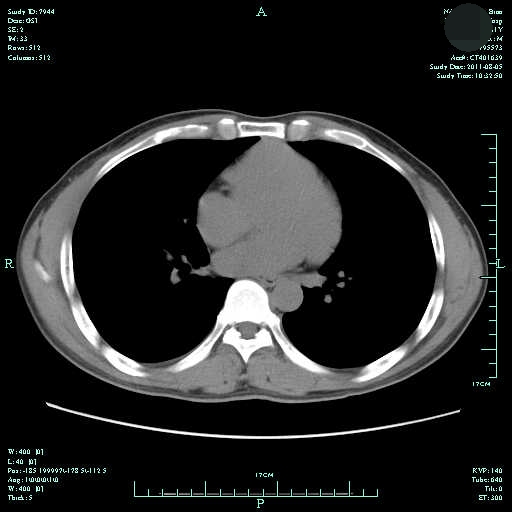

Supplement: Supplementary file 1 — 1. Plain scan CT. Plain scan CT of the esophagus showed the lesion located in the lower esophagus. The CT value is 16 Hu. 2. Enhanced CT. Enhanced CT of the esophagus showed the lesion located in the lower esophagus. The CT value is 38 Hu. 3. EUS. A large, whitish-yellow, translucent mass with a lustrous surface in the mid and distal esophagus, located 32 to 38 cm from the incisors. A honeycomb-like hypoechoic structure measuring 60 × 10 mm located in the submucosa with heterogenous echo pattern; the muscularis propria was intact. 4. EPMR. The esophageal mass was resected by endoscopic piecemeal mucosal resection (EPMR). 5. Pathology. Histology of the resected mass showing cystically dilated lymphatics in the surface squamous epithelium and in the submucosa. (hematoxylin and eosin [H&E], magnification ×200). [file 5747560.f1.zip › EPMR/Plain scan CT/exported0032_看图王.jpg]

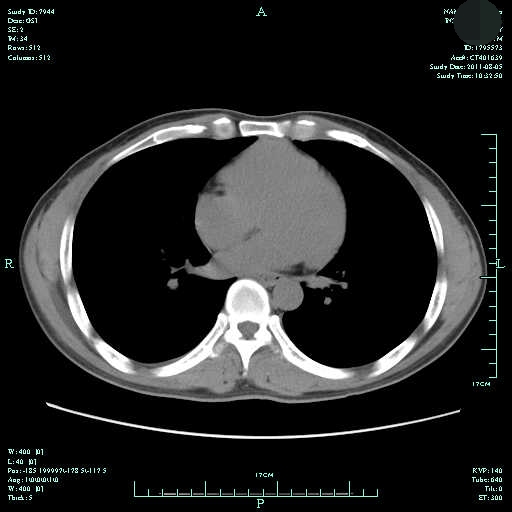

Supplement: Supplementary file 1 — 1. Plain scan CT. Plain scan CT of the esophagus showed the lesion located in the lower esophagus. The CT value is 16 Hu. 2. Enhanced CT. Enhanced CT of the esophagus showed the lesion located in the lower esophagus. The CT value is 38 Hu. 3. EUS. A large, whitish-yellow, translucent mass with a lustrous surface in the mid and distal esophagus, located 32 to 38 cm from the incisors. A honeycomb-like hypoechoic structure measuring 60 × 10 mm located in the submucosa with heterogenous echo pattern; the muscularis propria was intact. 4. EPMR. The esophageal mass was resected by endoscopic piecemeal mucosal resection (EPMR). 5. Pathology. Histology of the resected mass showing cystically dilated lymphatics in the surface squamous epithelium and in the submucosa. (hematoxylin and eosin [H&E], magnification ×200). [file 5747560.f1.zip › EPMR/Plain scan CT/exported0033_看图王.jpg]

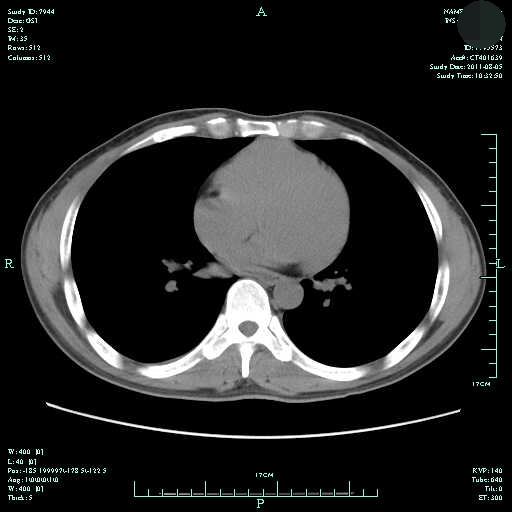

Supplement: Supplementary file 1 — 1. Plain scan CT. Plain scan CT of the esophagus showed the lesion located in the lower esophagus. The CT value is 16 Hu. 2. Enhanced CT. Enhanced CT of the esophagus showed the lesion located in the lower esophagus. The CT value is 38 Hu. 3. EUS. A large, whitish-yellow, translucent mass with a lustrous surface in the mid and distal esophagus, located 32 to 38 cm from the incisors. A honeycomb-like hypoechoic structure measuring 60 × 10 mm located in the submucosa with heterogenous echo pattern; the muscularis propria was intact. 4. EPMR. The esophageal mass was resected by endoscopic piecemeal mucosal resection (EPMR). 5. Pathology. Histology of the resected mass showing cystically dilated lymphatics in the surface squamous epithelium and in the submucosa. (hematoxylin and eosin [H&E], magnification ×200). [file 5747560.f1.zip › EPMR/Plain scan CT/exported0034_看图王.jpg]

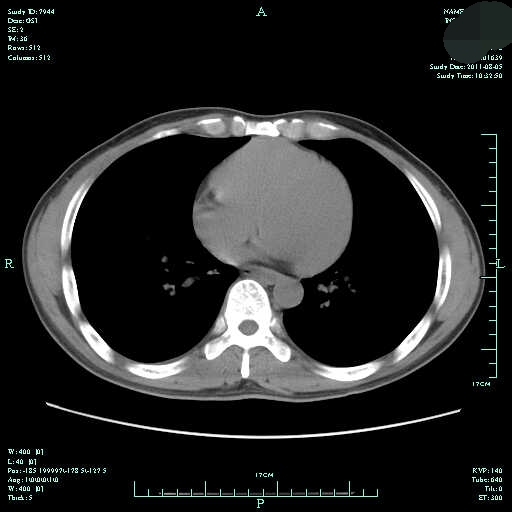

Supplement: Supplementary file 1 — 1. Plain scan CT. Plain scan CT of the esophagus showed the lesion located in the lower esophagus. The CT value is 16 Hu. 2. Enhanced CT. Enhanced CT of the esophagus showed the lesion located in the lower esophagus. The CT value is 38 Hu. 3. EUS. A large, whitish-yellow, translucent mass with a lustrous surface in the mid and distal esophagus, located 32 to 38 cm from the incisors. A honeycomb-like hypoechoic structure measuring 60 × 10 mm located in the submucosa with heterogenous echo pattern; the muscularis propria was intact. 4. EPMR. The esophageal mass was resected by endoscopic piecemeal mucosal resection (EPMR). 5. Pathology. Histology of the resected mass showing cystically dilated lymphatics in the surface squamous epithelium and in the submucosa. (hematoxylin and eosin [H&E], magnification ×200). [file 5747560.f1.zip › EPMR/Plain scan CT/exported0035_看图王.jpg]

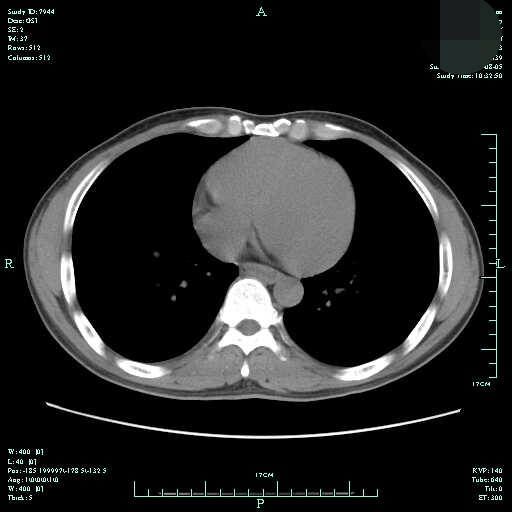

Supplement: Supplementary file 1 — 1. Plain scan CT. Plain scan CT of the esophagus showed the lesion located in the lower esophagus. The CT value is 16 Hu. 2. Enhanced CT. Enhanced CT of the esophagus showed the lesion located in the lower esophagus. The CT value is 38 Hu. 3. EUS. A large, whitish-yellow, translucent mass with a lustrous surface in the mid and distal esophagus, located 32 to 38 cm from the incisors. A honeycomb-like hypoechoic structure measuring 60 × 10 mm located in the submucosa with heterogenous echo pattern; the muscularis propria was intact. 4. EPMR. The esophageal mass was resected by endoscopic piecemeal mucosal resection (EPMR). 5. Pathology. Histology of the resected mass showing cystically dilated lymphatics in the surface squamous epithelium and in the submucosa. (hematoxylin and eosin [H&E], magnification ×200). [file 5747560.f1.zip › EPMR/Plain scan CT/exported0036_看图王.jpg]

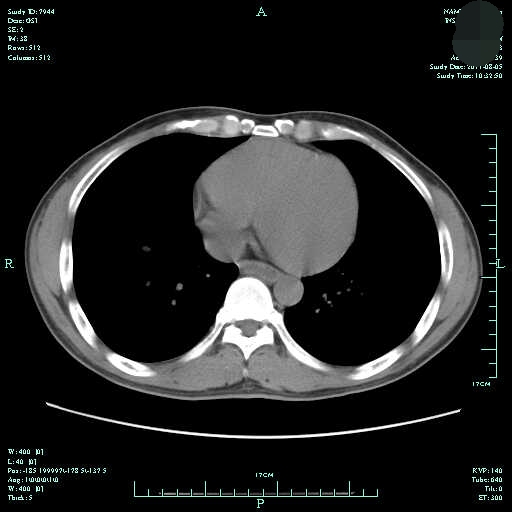

Supplement: Supplementary file 1 — 1. Plain scan CT. Plain scan CT of the esophagus showed the lesion located in the lower esophagus. The CT value is 16 Hu. 2. Enhanced CT. Enhanced CT of the esophagus showed the lesion located in the lower esophagus. The CT value is 38 Hu. 3. EUS. A large, whitish-yellow, translucent mass with a lustrous surface in the mid and distal esophagus, located 32 to 38 cm from the incisors. A honeycomb-like hypoechoic structure measuring 60 × 10 mm located in the submucosa with heterogenous echo pattern; the muscularis propria was intact. 4. EPMR. The esophageal mass was resected by endoscopic piecemeal mucosal resection (EPMR). 5. Pathology. Histology of the resected mass showing cystically dilated lymphatics in the surface squamous epithelium and in the submucosa. (hematoxylin and eosin [H&E], magnification ×200). [file 5747560.f1.zip › EPMR/Plain scan CT/exported0037_看图王.jpg]

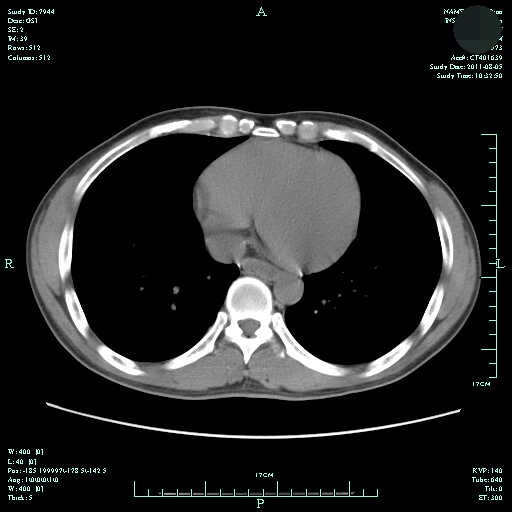

Supplement: Supplementary file 1 — 1. Plain scan CT. Plain scan CT of the esophagus showed the lesion located in the lower esophagus. The CT value is 16 Hu. 2. Enhanced CT. Enhanced CT of the esophagus showed the lesion located in the lower esophagus. The CT value is 38 Hu. 3. EUS. A large, whitish-yellow, translucent mass with a lustrous surface in the mid and distal esophagus, located 32 to 38 cm from the incisors. A honeycomb-like hypoechoic structure measuring 60 × 10 mm located in the submucosa with heterogenous echo pattern; the muscularis propria was intact. 4. EPMR. The esophageal mass was resected by endoscopic piecemeal mucosal resection (EPMR). 5. Pathology. Histology of the resected mass showing cystically dilated lymphatics in the surface squamous epithelium and in the submucosa. (hematoxylin and eosin [H&E], magnification ×200). [file 5747560.f1.zip › EPMR/Plain scan CT/exported0038_看图王.jpg]

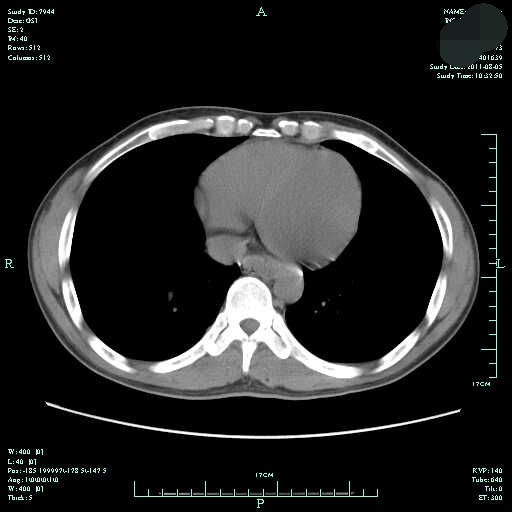

Supplement: Supplementary file 1 — 1. Plain scan CT. Plain scan CT of the esophagus showed the lesion located in the lower esophagus. The CT value is 16 Hu. 2. Enhanced CT. Enhanced CT of the esophagus showed the lesion located in the lower esophagus. The CT value is 38 Hu. 3. EUS. A large, whitish-yellow, translucent mass with a lustrous surface in the mid and distal esophagus, located 32 to 38 cm from the incisors. A honeycomb-like hypoechoic structure measuring 60 × 10 mm located in the submucosa with heterogenous echo pattern; the muscularis propria was intact. 4. EPMR. The esophageal mass was resected by endoscopic piecemeal mucosal resection (EPMR). 5. Pathology. Histology of the resected mass showing cystically dilated lymphatics in the surface squamous epithelium and in the submucosa. (hematoxylin and eosin [H&E], magnification ×200). [file 5747560.f1.zip › EPMR/Plain scan CT/exported0039_看图王.jpg]

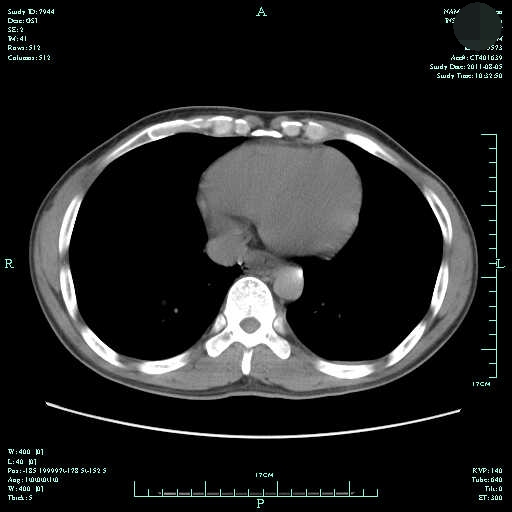

Supplement: Supplementary file 1 — 1. Plain scan CT. Plain scan CT of the esophagus showed the lesion located in the lower esophagus. The CT value is 16 Hu. 2. Enhanced CT. Enhanced CT of the esophagus showed the lesion located in the lower esophagus. The CT value is 38 Hu. 3. EUS. A large, whitish-yellow, translucent mass with a lustrous surface in the mid and distal esophagus, located 32 to 38 cm from the incisors. A honeycomb-like hypoechoic structure measuring 60 × 10 mm located in the submucosa with heterogenous echo pattern; the muscularis propria was intact. 4. EPMR. The esophageal mass was resected by endoscopic piecemeal mucosal resection (EPMR). 5. Pathology. Histology of the resected mass showing cystically dilated lymphatics in the surface squamous epithelium and in the submucosa. (hematoxylin and eosin [H&E], magnification ×200). [file 5747560.f1.zip › EPMR/Plain scan CT/exported0040_看图王.jpg]

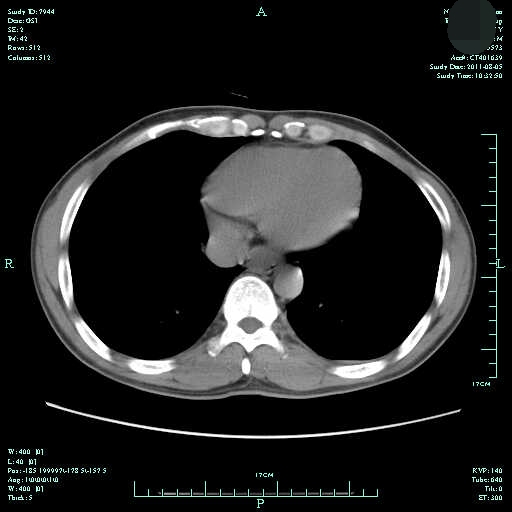

Supplement: Supplementary file 1 — 1. Plain scan CT. Plain scan CT of the esophagus showed the lesion located in the lower esophagus. The CT value is 16 Hu. 2. Enhanced CT. Enhanced CT of the esophagus showed the lesion located in the lower esophagus. The CT value is 38 Hu. 3. EUS. A large, whitish-yellow, translucent mass with a lustrous surface in the mid and distal esophagus, located 32 to 38 cm from the incisors. A honeycomb-like hypoechoic structure measuring 60 × 10 mm located in the submucosa with heterogenous echo pattern; the muscularis propria was intact. 4. EPMR. The esophageal mass was resected by endoscopic piecemeal mucosal resection (EPMR). 5. Pathology. Histology of the resected mass showing cystically dilated lymphatics in the surface squamous epithelium and in the submucosa. (hematoxylin and eosin [H&E], magnification ×200). [file 5747560.f1.zip › EPMR/Plain scan CT/exported0041_看图王.jpg]

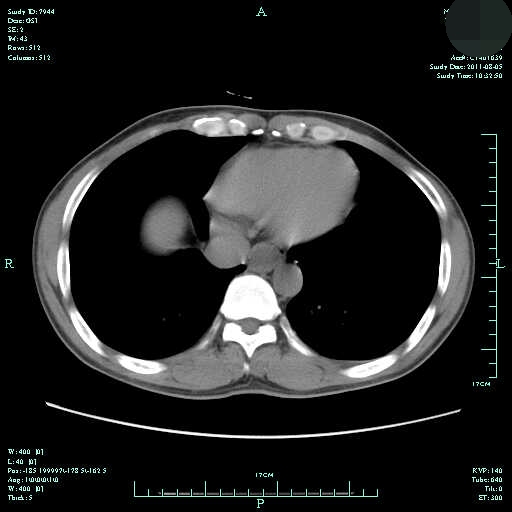

Supplement: Supplementary file 1 — 1. Plain scan CT. Plain scan CT of the esophagus showed the lesion located in the lower esophagus. The CT value is 16 Hu. 2. Enhanced CT. Enhanced CT of the esophagus showed the lesion located in the lower esophagus. The CT value is 38 Hu. 3. EUS. A large, whitish-yellow, translucent mass with a lustrous surface in the mid and distal esophagus, located 32 to 38 cm from the incisors. A honeycomb-like hypoechoic structure measuring 60 × 10 mm located in the submucosa with heterogenous echo pattern; the muscularis propria was intact. 4. EPMR. The esophageal mass was resected by endoscopic piecemeal mucosal resection (EPMR). 5. Pathology. Histology of the resected mass showing cystically dilated lymphatics in the surface squamous epithelium and in the submucosa. (hematoxylin and eosin [H&E], magnification ×200). [file 5747560.f1.zip › EPMR/Plain scan CT/exported0042_看图王.jpg]

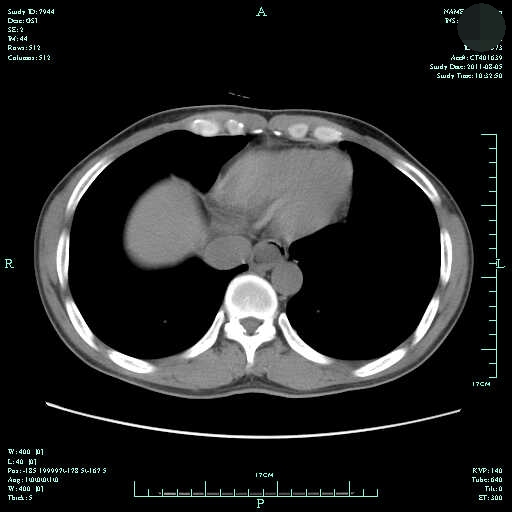

Supplement: Supplementary file 1 — 1. Plain scan CT. Plain scan CT of the esophagus showed the lesion located in the lower esophagus. The CT value is 16 Hu. 2. Enhanced CT. Enhanced CT of the esophagus showed the lesion located in the lower esophagus. The CT value is 38 Hu. 3. EUS. A large, whitish-yellow, translucent mass with a lustrous surface in the mid and distal esophagus, located 32 to 38 cm from the incisors. A honeycomb-like hypoechoic structure measuring 60 × 10 mm located in the submucosa with heterogenous echo pattern; the muscularis propria was intact. 4. EPMR. The esophageal mass was resected by endoscopic piecemeal mucosal resection (EPMR). 5. Pathology. Histology of the resected mass showing cystically dilated lymphatics in the surface squamous epithelium and in the submucosa. (hematoxylin and eosin [H&E], magnification ×200). [file 5747560.f1.zip › EPMR/Plain scan CT/exported0043_看图王.jpg]

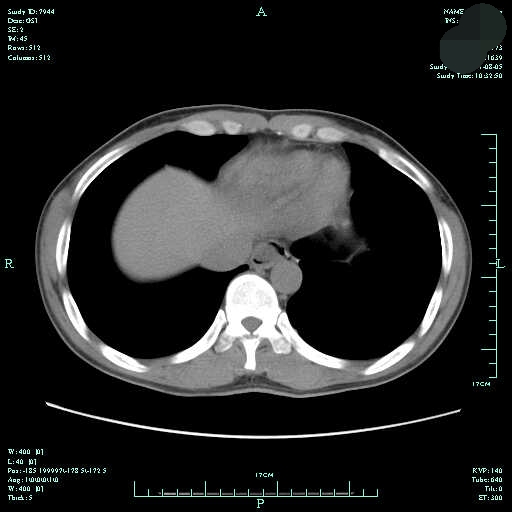

Supplement: Supplementary file 1 — 1. Plain scan CT. Plain scan CT of the esophagus showed the lesion located in the lower esophagus. The CT value is 16 Hu. 2. Enhanced CT. Enhanced CT of the esophagus showed the lesion located in the lower esophagus. The CT value is 38 Hu. 3. EUS. A large, whitish-yellow, translucent mass with a lustrous surface in the mid and distal esophagus, located 32 to 38 cm from the incisors. A honeycomb-like hypoechoic structure measuring 60 × 10 mm located in the submucosa with heterogenous echo pattern; the muscularis propria was intact. 4. EPMR. The esophageal mass was resected by endoscopic piecemeal mucosal resection (EPMR). 5. Pathology. Histology of the resected mass showing cystically dilated lymphatics in the surface squamous epithelium and in the submucosa. (hematoxylin and eosin [H&E], magnification ×200). [file 5747560.f1.zip › EPMR/Plain scan CT/exported0044_看图王.jpg]

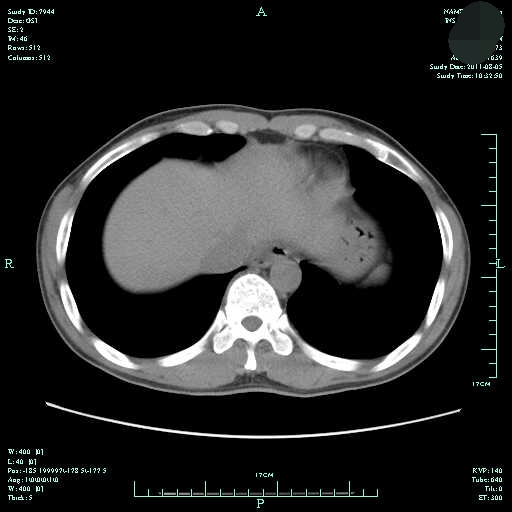

Supplement: Supplementary file 1 — 1. Plain scan CT. Plain scan CT of the esophagus showed the lesion located in the lower esophagus. The CT value is 16 Hu. 2. Enhanced CT. Enhanced CT of the esophagus showed the lesion located in the lower esophagus. The CT value is 38 Hu. 3. EUS. A large, whitish-yellow, translucent mass with a lustrous surface in the mid and distal esophagus, located 32 to 38 cm from the incisors. A honeycomb-like hypoechoic structure measuring 60 × 10 mm located in the submucosa with heterogenous echo pattern; the muscularis propria was intact. 4. EPMR. The esophageal mass was resected by endoscopic piecemeal mucosal resection (EPMR). 5. Pathology. Histology of the resected mass showing cystically dilated lymphatics in the surface squamous epithelium and in the submucosa. (hematoxylin and eosin [H&E], magnification ×200). [file 5747560.f1.zip › EPMR/Plain scan CT/exported0045_看图王.jpg]

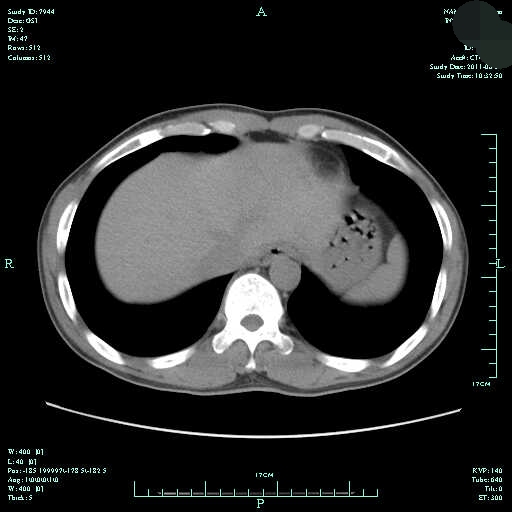

Supplement: Supplementary file 1 — 1. Plain scan CT. Plain scan CT of the esophagus showed the lesion located in the lower esophagus. The CT value is 16 Hu. 2. Enhanced CT. Enhanced CT of the esophagus showed the lesion located in the lower esophagus. The CT value is 38 Hu. 3. EUS. A large, whitish-yellow, translucent mass with a lustrous surface in the mid and distal esophagus, located 32 to 38 cm from the incisors. A honeycomb-like hypoechoic structure measuring 60 × 10 mm located in the submucosa with heterogenous echo pattern; the muscularis propria was intact. 4. EPMR. The esophageal mass was resected by endoscopic piecemeal mucosal resection (EPMR). 5. Pathology. Histology of the resected mass showing cystically dilated lymphatics in the surface squamous epithelium and in the submucosa. (hematoxylin and eosin [H&E], magnification ×200). [file 5747560.f1.zip › EPMR/Plain scan CT/exported0046_看图王.jpg]
